# Supplementary material for: Social media does not elicit a physiological stress response as measured by heart rate and salivary cortisol over 20-minute sessions of cell phone use
Source: PLoS One. 2024 Apr 3;19(4):e0298553. doi: 10.1371/journal.pone.0298553 (PMC10990243; doi:10.1371/journal.pone.0298553)
Supplement: S1 Appendix — (DOCX) [file pone.0298553.s001.docx]

**S1 Appendix. YouTube videos included in our non-evocative playlist**

**Link to non-evocative playlist**

<https://tinyurl.com/CWI-YOUTUBE>

**List of and links to individual videos within non-evocative playlist**

Neutral Emotion Pictures Video: https://www.youtube.com/watch?v=7luz5pQ0gko&list=PLsU6oWbuunwGAAgI0Pnt4C25gVvsEgNWr&index=1

Kids Try 100 Years of the Most Expensive Foods: https://www.youtube.com/watch?v=7LjC-nUPrNg&list=PLsU6oWbuunwGAAgI0Pnt4C25gVvsEgNWr&index=2

29 COMMERCIAL TRICKS WE ALWAYS BELIEVED: https://www.youtube.com/watch?v=gQzcno9r1OQ&list=PLsU6oWbuunwGAAgI0Pnt4C25gVvsEgNWr&index=3

Crushed by a Giant 6ft Water Balloon - The Slow Mo Guys 4K: https://www.youtube.com/watch?v=fZI5oZ-1NdA&list=PLsU6oWbuunwGAAgI0Pnt4C25gVvsEgNWr&index=5&ab_channel=TheSlowMoGuys

Gravity Visualized: https://www.youtube.com/watch?v=MTY1Kje0yLg&list=PLsU6oWbuunwGAAgI0Pnt4C25gVvsEgNWr&index=5

Things You Did Not Know The Use Of: https://www.youtube.com/watch?v=86ltPV7Ku0A&list=PLsU6oWbuunwGAAgI0Pnt4C25gVvsEgNWr&index=6

Things You're Consuming Wrong: https://www.youtube.com/watch?v=aVWJMcOptR8&list=PLsU6oWbuunwGAAgI0Pnt4C25gVvsEgNWr&index=7

10 Secrets Hidden Inside Famous Logos: https://www.youtube.com/watch?v=xSVYhd1iBbM&list=PLsU6oWbuunwGAAgI0Pnt4C25gVvsEgNWr&index=8
