# Supplementary material for: Social media does not elicit a physiological stress response as measured by heart rate and salivary cortisol over 20-minute sessions of cell phone use
Source: PLoS One. 2024 Apr 3;19(4):e0298553. doi: 10.1371/journal.pone.0298553 (PMC10990243; doi:10.1371/journal.pone.0298553)
Supplement: S2 Appendix — (DOCX) [file pone.0298553.s002.docx]

**S2 Appendix. Psychological scales**

**Intensity of social media use**

Our participants selected either the Facebook or Instagram Intensity Scale to measure the degree to which they engage with their chosen social media platform. The scales for each platform are approximately equivalent; the language in the Facebook Intensity Scale, created by Ellison et al. (55), was minimally modified by Teo and Collinson (56) to apply to Instagram as the Instagram Intensity Scale. The Facebook and Instagram intensity scales measure participants’ engagement with these platforms with respect to their number of “friends,” daily time engagement, emotional investment, and extent to which one’s daily activities are integrated with the platform. The Facebook and Instagram Intensity Scales consist of six statements in which participants choose an answer ranging from 1) *strongly disagree* to 5) *strongly agree.* There are three open-ended questions regarding the number of social media friends and followers and the frequency with which they checked their social media feeds within the past week. Responses to these three questions are collapsed into an ordinal scale that also ranges from 1 to 5. Responses on this scale were summed for each participant, with higher scores indicating greater intensity of social media use. One participant left two items blank on the Facebook Intensity Scale; these two items were imputed with the overall mean for these items. These imputations allowed all participants to have a response on this scale.

**Self-esteem scale**

Self-esteem was measured with the Rosenberg Self-Esteem Scale (57) which quantifies feelings of self-worth and self-acceptance. The scale contains 10 statements about feelings and attitudes towards one’s self. Responses to statements are made on a four-point scale anchored by *strongly agree* and *strongly disagree*. After accounting for items with reversed scoring, a summed self-esteem score was calculated for each participant. Higher numbers indicate greater self-esteem. While subscales are considered reliable and valid for this instrument, we used the overall score. One participant neglected a single item on the Self-Esteem Scale, and this was imputed using the participant’s response to the other item on the same subscale. This imputation allowed all participants to have a response on this scale.

**Social comparison scale**

The Social Comparison Scale (58) assesses self-perception of social rank, relative attractiveness, and acceptance within one’s social group. The scale used in our study was modified by Teo & Collinson (56) to pertain specifically to social media. This scale consists of 10 incomplete sentences in which participants select a relative number between two bipolar constructs representing their self-perception. For instance, a participant using Facebook would select a number between 1 and 10 in response to the statement “When I compare myself to others on Facebook, I feel *Inferior* 1 2 3 4 5 6 7 8 9 10 *Superior*.” A sum of quantified responses is calculated as the measure outcome, with higher scores indicating that the participant perceives a more favorable social position for themselves, compared to others within their social group.

**Perceived stress scale**

Participants’ self-assessment of stress was measured with The Perceived Stress Scale (59). This scale consists of 14 questions pertaining to the participants’ thoughts and feelings during the previous month. Response options are made on a four-point scale ranging from never to very often. After accounting for items with reversed scoring, a sum of scores was calculated for each participant, with higher scores indicating higher stress. To avoid the possibility that thoughts of stress prompted by this scale could influence responses on other scales, this was the last scale administered in our study.
