# Supplementary material for: Social media does not elicit a physiological stress response as measured by heart rate and salivary cortisol over 20-minute sessions of cell phone use
Source: PLoS One. 2024 Apr 3;19(4):e0298553. doi: 10.1371/journal.pone.0298553 (PMC10990243; doi:10.1371/journal.pone.0298553)
Supplement: Attachment — Submitted filename: PONE-D-23-15507-rev - Copy.pdf [file pone.0298553.s004.pdf]

PLOS ONE

Routine Use of Social Media Does Not Provoke a Physiological Stress Response

--Manuscript Draft--

**Manuscript Number:**	PONE-D-23-15507
**Article Type:**	Research Article
**Full Title:**	Routine Use of Social Media Does Not Provoke a Physiological Stress Response
**Short Title:**	Social media and physiological stress response
**Corresponding Author:**	Suzanne Oppenheimer, Ph.D.<br>College of Western Idaho - Ada County Campus<br>Boise, ID UNITED STATES
**Keywords:**	Stress; cortisol; heart rate; social media
**Abstract:**	The pervasive use of social media has raised concerns about its potential detrimental health effects. We examined physiological indicators of stress associated with social media use and investigated possible moderating influences of sex, age, and psychological parameters. We measured heart rate and cortisol in 59 subjects ranging in age from 13 to 55 during and after two cell phone treatments: social media use and a controlled YouTube playlist. To disentangle the effects of cell phone treatment from order of treatment, we used a crossover design in which participants were randomized to treatment order. Our study uncovered a significant period effect suggesting that both heart rate and cortisol decreased over the duration of our experiment, irrespective of the type of cell phone activity or the order of treatments. There was no indication that age, sex, habits of social media use, or psychometric parameters moderated the physiological response to cell phone activities. Our data suggest that smartphone use does not provoke a physiological stress response.
**Order of Authors:**	Suzanne Oppenheimer, Ph.D.
	Laura Bond
	Charity Smith
**Additional Information:**	
**Question**	**Response**
**Financial Disclosure**<br><br>Enter a financial disclosure statement that describes the sources of funding for the work included in this submission. Review the submission guidelines for detailed requirements. View published research articles from *PLOS ONE* for specific examples.<br><br>This statement is required for submission and **will appear in the published article** if the submission is accepted. Please make sure it is accurate.	SO,LB,CS: This research was made possible by Institutional Development Awards (IDeA) from the National Institute of General Medical Sciences of the National Institutes of Health under Grant #P20GM103408.<br>LB: Additional support was offered by Grants # 1U54GM104944, P20GM109095, and 1C06RR020533. We also acknowledge support from The Biomolecular Research Center at Boise State, BSU-Biomolecular Research Center, RRID:SCR_019174, with funding from the National Science Foundation, Grants #0619793 and #0923535; the M. J. Murdock Charitable Trust; Lori and Duane Stueckle, and the Idaho State Board of Education.<br><br>The funders had no role in study design, data collection and analysis, decision to publish, or preparation of the manuscript.

Powered by Editorial Manager® and ProduXion Manager® from Aries Systems Corporation

**Unfunded studies**<br>Enter: *The author(s) received no specific funding for this work.*<br><br>**Funded studies**<br>Enter a statement with the following details:<br>• Initials of the authors who received each award<br>• Grant numbers awarded to each author<br>• The full name of each funder<br>• URL of each funder website<br>• Did the sponsors or funders play any role in the study design, data collection and analysis, decision to publish, or preparation of the manuscript?<br>• **NO** - Include this sentence at the end of your statement: *The funders had no role in study design, data collection and analysis, decision to publish, or preparation of the manuscript.*<br>• **YES** - Specify the role(s) played.<br><br>* typeset	
**Competing Interests**<br><br>Use the instructions below to enter a competing interest statement for this submission. On behalf of all authors, disclose any competing interests that could be perceived to bias this work—acknowledging all financial support and any other relevant financial or non-financial competing interests.<br><br>This statement is required for submission and **will appear in the published article** if the submission is accepted. Please make sure it is accurate and that any funding sources listed in your Funding Information later in the submission form are also declared in your Financial Disclosure statement.<br><br>View published research articles from *PLOS ONE* for specific examples.	The authors have declared that no competing interests exist.

**NO authors have competing interests**<br><br>Enter: *The authors have declared that no competing interests exist.*<br><br>**Authors with competing interests**<br><br>Enter competing interest details beginning with this statement:<br><br>*I have read the journal's policy and the authors of this manuscript have the following competing interests: [insert competing interests here]*<br><br>* typeset	
**Ethics Statement**<br><br>Enter an ethics statement for this submission. This statement is required if the study involved:<br><br>• Human participants<br>• Human specimens or tissue<br>• Vertebrate animals or cephalopods<br>• Vertebrate embryos or tissues<br>• Field research<br><br>Write "N/A" if the submission does not require an ethics statement.<br><br>General guidance is provided below. Consult the submission guidelines for detailed instructions. **Make sure that all information entered here is included in the Methods section of the manuscript.**	Ethics Statement<br>The study protocol was approved by the Institutional Review Board of The College of Idaho (1819-CWI00039.3). Adult subjects signed written informed consent forms, and those under the age of 18 years old obtained written consent from a parent, guardian, or legally authorized representative. Participants self-reported their biological sex and age on surveys.

**Format for specific study types**<br><br>**Human Subject Research (involving human participants and/or tissue)**<br>• Give the name of the institutional review board or ethics committee that approved the study<br>• Include the approval number and/or a statement indicating approval of this research<br>• Indicate the form of consent obtained (written/oral) or the reason that consent was not obtained (e.g. the data were analyzed anonymously)<br><br>**Animal Research (involving vertebrate animals, embryos or tissues)**<br>• Provide the name of the Institutional Animal Care and Use Committee (IACUC) or other relevant ethics board that reviewed the study protocol, and indicate whether they approved this research or granted a formal waiver of ethical approval<br>• Include an approval number if one was obtained<br>• If the study involved *non-human primates*, add *additional details* about animal welfare and steps taken to ameliorate suffering<br>• If anesthesia, euthanasia, or any kind of animal sacrifice is part of the study, include briefly which substances and/or methods were applied<br><br>**Field Research**<br><br>Include the following details if this study involves the collection of plant, animal, or other materials from a natural setting:<br>• Field permit number<br>• Name of the institution or relevant body that granted permission	
**Data Availability**<br><br>Authors are required to make all data underlying the findings described fully available, without restriction, and from the time of publication. PLOS allows rare exceptions to address legal and ethical concerns. See the PLOS Data Policy and FAQ for detailed information.	Yes - all data are fully available without restriction

A Data Availability Statement describing where the data can be found is required at submission. Your answers to this question constitute the Data Availability Statement and **will be published in the article**, if accepted.<br><br>**Important:** Stating 'data available on request from the author' is not sufficient. If your data are only available upon request, select 'No' for the first question and explain your exceptional situation in the text box.<br><br>Do the authors confirm that all data underlying the findings described in their manuscript are fully available without restriction?	
**Describe where the data may be found in full sentences. If you are copying our sample text, replace any instances of XXX with the appropriate details.**<br><br>• If the data are **held or will be held in a public repository**, include URLs, accession numbers or DOIs. If this information will only be available after acceptance, indicate this by ticking the box below. For example: *All XXX files are available from the XXX database (accession number(s) XXX, XXX.)*.<br>• If the data are all contained **within the manuscript and/or Supporting Information files**, enter the following: *All relevant data are within the manuscript and its Supporting Information files.*<br>• If neither of these applies but you are able to provide **details of access elsewhere**, with or without limitations, please do so. For example:<br><br>*Data cannot be shared publicly because of [XXX]. Data are available from the XXX Institutional Data Access / Ethics Committee (contact via XXX) for researchers who meet the criteria for access to confidential data.*<br><br>*The data underlying the results presented in the study are available from (include the name of the third party*	Accession numbers and/or DOIs will be made available after acceptance.<br><br>All XXX files are available from the XXX database (accession number(s) XXX, XXX.)

and contact information or URL).<br>• This text is appropriate if the data are owned by a third party and authors do not have permission to share the data.<br><br>* typeset	
Additional data availability information:	Tick here if the URLs/accession numbers/DOIs will be available only after acceptance of the manuscript for publication so that we can ensure their inclusion before publication.

Powered by Editorial Manager® and ProduXion Manager® from Aries Systems Corporation

Manuscript

Click here to access/download;Manuscript;PLOS_submission_May2023.docx

Routine Use of Social Media Does Not Provoke a Physiological Stress Response

Suzanne Oppenheimer[a] (corresponding author), Laura Bond[b], Charity Smith[a]

[a]Biological Sciences, College of Western Idaho, Ada County Campus Pintail Center, 1360 S. Eagle Flight Way, Boise, ID 83709, USA, suzanneoppenheimer@cwi.edu; charitysmith826@u.boisestate.edu
[b]Biomolecular Research Center, Boise State University, Mathematics Building Room 225, 1910 University Drive, Boise, Idaho 83725, USA, lbond@boisestate.edu

Abstract

The pervasive use of social media has raised concerns about its potential detrimental health effects. We examined physiological indicators of stress associated with social media use and investigated possible moderating influences of sex, age, and psychological parameters. We measured heart rate and cortisol in 59 subjects ranging in age from 13 to 55 during and after two cell phone treatments: social media use and a controlled YouTube playlist. To disentangle the effects of cell phone treatment from order of treatment, we used a crossover design in which participants were randomized to treatment order. Our study uncovered a significant period effect suggesting that both heart rate and cortisol decreased over the duration of our experiment, irrespective of the type of cell phone activity or the order of treatments. There was no indication that age, sex, habits of social media use, or psychometric parameters moderated the physiological response to cell phone activities. Our data suggest that smartphone use does not provoke a physiological stress response.

Keywords: stress, cortisol, heart rate, social media

Introduction

With widespread use of individually-accessible screens (e.g. smart phones, tablets, and computers), many people have expressed concern over excessive use; for example, the American Academy of Pediatrics (2016), Britain's Chief Medical Officer (Great Britain 2019), and eminent psychologist Jean Twenge (2017) have issued warnings against overuse of smartphones by children. While screens are the instrument, social media is one of the elements often implicated in the dangers associated with screen time. Unlike in-person social interactions, which are often associated with higher psychological well-being (Baumeister & Leary 1995, Diener et al. 1999), excessive social media interactions have been linked to poor psychological well-being (Lin et al. 2016, Shakya & Christakis 2017, Twenge et al. 2018).

Some studies have suggested that social media use may provoke psychosocial stress (Affi et al. 2018, Rauch et al. 2014, Rus and Tiemensma 2017), and social media use has frequently been linked to conditions such as anxiety and depression (e.g. Barry et al. 2017, Ehrenreich & Underwood 2016, Woods & Scott 2016). Fear of missing out, the worry that one may miss out on important interpersonal interactions or events (Wang et al. 2018), seems to both drive excessive use of social media and contribute to depression and anxiety associated with its use (Dempsey et al. 2019, Przybylski et al. 2013). In some cases, social media promotes low self-esteem, poor body image, and envy (Kelly et al. 2018, Verduyn et al. 2017); such feelings are thought to be evoked when social media users evaluate their social and personal worth by comparing themselves to others who have curated and/or enhanced an idealistic online image (Hawes et al. 2020). Poor sleep quality, which has been linked to computer, internet, and social media use (Woods & Scott 2016), is also thought to contribute to depression,

anxiety, and low self-esteem (Alfano et al. 2009, Fredriksen et al. 2004). Taken together, the body of literature suggests that multiple factors associated with social media create psychosocial stress.

Psychological stress is known to stimulate a physiological stress response, mediated by the endocrine and nervous systems (Jezova et al. 2004, Lupis et al. 2014, Owens et al. 2017). While the stress response helps maintain physiologic balance in the face of sudden physiological or psychological perturbations (Sapolsky 2004), chronic activation of this response can result in an increased allostatic load and disease (Juster et al. 2010); for example, stress has been linked to depression, cardiovascular disease, and type 2 diabetes (Bhagwagar et al. 2005, McEwen 1998, Rosmond et al. 2003, respectively). If the use of social media causes psychosocial stress and subsequent activation of the physiological stress response, then protracted use could have deleterious consequences for both psychological and physical health. Several studies have examined the relationship between physiological variables and social media use, but the findings are equivocal (Table 1).

Table 1. Summary of key findings in studies examining the relationship between physiological variables and social media. HR=heart rate, BP=blood pressure, MAP=mean arterial pressure

**Physiological Variables**	**Key Findings**	**Reference**
cortisol	diurnal cortisol levels positively associated with number of Facebook friends and negatively associated with Facebook peer interactions	Morin-Major et al. 2016
cortisol	Facebook vs. control treatment following Trier Social Stress Test (TSST):<br>-greater sustained cortisol following Facebook use	Rus & Tiemensma 2017
cortisol	adolescents who report more frequent use of social media have a greater cortisol awakening response	Afifi et al. 2018
cortisol<br>heart rate (HR)<br>diastolic/systolic blood pressure (BP)	Facebook vs. control treatment followed by Trier Social Stress Test (TSST):<br>-lower increase in systolic BP in Facebook than control<br>-no difference in HR recovery, diastolic BP, or salivary cortisol	Rus & Tiemensma 2018
mean arterial pressure (MAP)<br>heart rate	social media use following TSST facilitated recovery of MAP & HR	Johnshoy et al. 2020
cortisol	salivary cortisol positively associated with social media usage and addiction	Shafi et al. 2021

Our study directly evaluates the relationship between routine social media use and the physiological stress response and indirectly takes into account the potential moderating effects of age, sex, and psychosocial factors. We hypothesized that social media use would impart some degree of psychosocial stress that would activate the physiological stress response more so than our control treatment (non-evocative You-tube playlist). To assess the physiological response, we measured salivary cortisol to quantify activity of the hypothalamic-pituitary-adrenal (HPA) axis and heart rate to assess activation of the sympathetic nervous system.

Material and methods

Ethics Statement

The study protocol was approved by the Institutional Review Board of The College of Idaho (1819-CWI00039.3). Adult subjects signed written informed consent forms, and those under the age of 18 years old obtained written consent from a parent, guardian, or legally authorized representative. Participants self-reported their biological sex and age on surveys.

Participants

Participants were recruited in July 2019 using fliers, internet announcements, and direct oral solicitation, primarily directed toward faculty, students, and staff at the College of Western Idaho. The study was broadly open to adults, but individuals under 18 years were encouraged to join with parental consent. Pregnant women and those with Cushing's syndrome were excluded. Subjects included in cortisol analyses had not consumed alcohol within 12 hours and had not eaten a meal within 60 minutes (identified through questionnaires). Because our cortisol assay exhibited low cross-reactivity with steroids commonly used in hormonal contraceptives (Salimetrics 2015), women on birth control were not excluded from our analyses (12.5% of females reported the use of hormonal contraceptives).

Participants included 40 women and 19 men, ranging in age from 13 to 55 with a mean of 25.8 years and a standard deviation of 11.5 years. Twelve participants were under 18 (5 women and 7 men). The study was conducted over three days, with 15 participants on the first day, 21 on the second day, and 23 on the third day. On a given day, all participated simultaneously and were randomly assigned to each experimental group order. Heart rate data were not available for six participants. The cortisol data from three participants were not used in the analysis because they ate within the hour prior to the study. Cortisol values from two individuals at a single time point were unavailable for analysis due to processing issues. Otherwise, all individuals completed the entire study, including completing all questionnaires.

Overall Procedures

Participants were asked to attend a single experimental session on one of three testing days at 12:30PM and to refrain from eating at least one hour prior to testing. Participants were assigned a random number, and all collected data were linked to the participants' numbers and not their names or other identifiers. Subjects signed informed consent forms and were randomized into one of two groups. We employed a two-period crossover design (Jones & Kenward 2015, Millikin & Johnson 2009) to evaluate the physiological response to social media relative to non-evocative content on YouTube. With this approach, all participants experienced both experimental conditions but the order in which they experienced them was randomized (Social Media first, or YouTube playlist first). Data were analyzed in a repeated measures framework so that each participant was compared to themselves, and the two experimental orders ensured that a period effect would not be misidentified as a treatment effect.

On the day of testing, participants entered the testing room and were fitted with arm-band heart rate monitors which continuously collected data until the conclusion of our protocol. During the pre-test 'Baseline' Period, participants sat quietly (without interacting with their cell phones or each other) for 5-15 minutes (depending on order of entry) and waited for instructions (Figure 1). Subjects were briefed on the study protocol and baseline saliva samples were collected.

During the first experimental period, “T1,” participants were asked to use their personal cell phones to view either a non-evocative YouTube playlist (*YouTube* treatment) or to browse their Facebook or Instagram accounts (*Social Media* treatment). Participants wore headphones during cell phone activities to avoid distracting one another. Because we were interested in the effect of participants’ typical social media experiences, rather than the effect of a particular platform, we asked participants to select their customary social media platforms from among the two most popular: Instagram and Facebook (Pew Research Center 2018). The non-evocative YouTube playlist was used as a negative control and consisted of a selection of videos such as *Neutral Emotion Pictures Video* and *Things You Did Not Know the Use Of*; when interacting with this playlist, participants engaged in a similar scrolling and viewing behavior as they did when engaging with their social media accounts. This cell phone activity lasted 20 minutes and was followed by a 5-minute interim period, during which a second saliva sample was taken to measure the cortisol response during the first experimental period. Participants did not interact with their cell phones or each other during this interim period.

During the second experimental period, “T2,” subjects were again asked to use their personal cell phones for 20 minutes to view either *YouTube* or *Social Media*; this selection alternated from the subject’s first treatment, so that each subject viewed both treatments but in random order. Following this 20-minute cell phone activity, a third saliva sample (“Final”) was collected, after which participants completed an online demographic and psychological survey via google forms (see Psychological Measures). We chose 20-minute experimental periods because several studies have shown that salivary cortisol peaks 20 minutes after a psychological stressor (Becker & Rohleder 2019, Engert et al. 2011, Kudielka et al. 2004). Heart rate monitors were collected from participants after they completed the questionnaires. Figure 1 provides a schematic of the overall study design, indicating the study periods and the abbreviations used throughout this article.

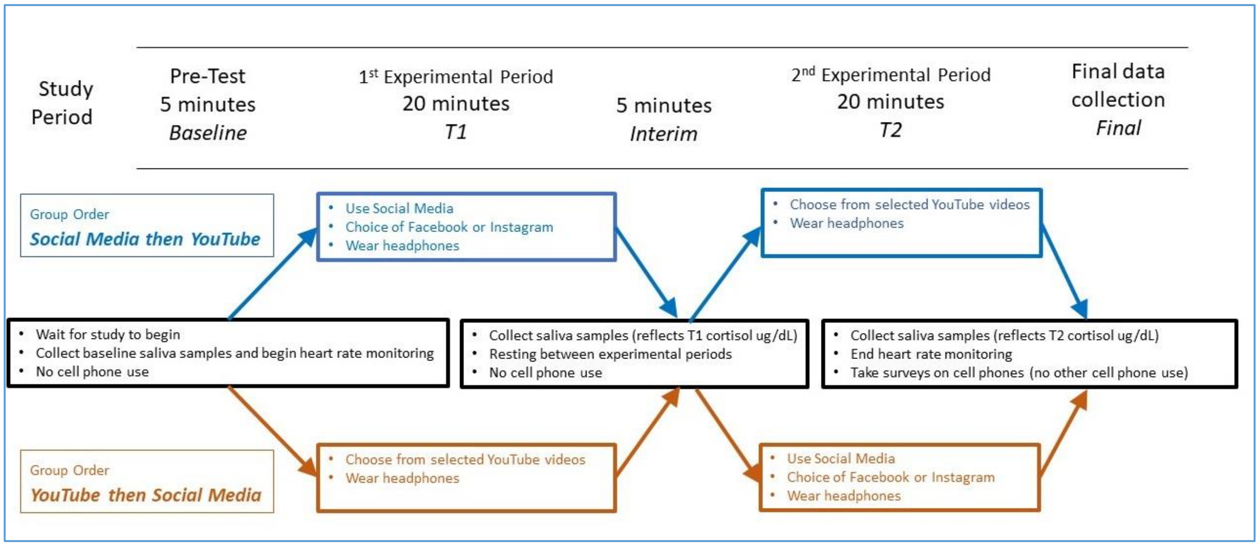

Figure 1. Schematic of study design. Study periods are Baseline (the five minutes prior to the first cell phone activity), T1 and T2 identify the two 20-minute experimental periods during which cell phones were used for Social Media or YouTube, Interim identifies the five-minute period between the two experimental periods, and Final reflects the period of the final saliva collection and when participants filled out the survey. The saliva sample collected in the Final period reflects the response from the second experimental period.

Physiological Measures

To evaluate subjects' physiological responses, both heart rate and salivary cortisol levels were measured. Subjects wore Polar OH1 (Polar Electro Oy, Finland) optical heart rate sensors around their upper arm to continuously record heart rate for the duration of the experiment. These sensors utilize photoplethysmography technology to determine heart rate and have a sampling rate of 50Hz.

Heart rate was continuously measured from the time when the monitors were placed on the participants' arms. Using time records, heart rate data were encoded to match the study periods described above. We calculated the average heart rate, the median heart rate, and the 95th percentile heart rate of each individual for all four periods. Additionally, for the two experimental periods, we calculated the mean heart rate of the first and last five minutes (activity minutes 1-5 and 15-20). The relative differences between heart rate and our resulting conclusions did not vary across any of the five measures, so we present only the mean heart rate results for simplicity.

Subjects provided saliva samples before the first cell phone treatment and immediately after each of the two treatments. To collect saliva, subjects placed absorbent swabs (SalivaBio Oral Swabs, Salimetrics; State College, PA) in their mouths for 2 minutes and then removed and sealed the swabs in purposely designed tubes. Swabs were centrifuged and the resulting saliva samples (approximately 1ml) were frozen at -20°C for up to one week before analysis. Cortisol was measured using a commercially available enzyme immunoassay kit (Salimetrics; State College, PA). The lower limit of sensitivity for this assay was 0.007µg/dl, and the inter-assay variation was 5.6% and 5.4% for high and low controls, respectively. Intra-assay variation ranged from 2.3-5.4%. All indicate acceptable limits of variation. The cortisol antiserum cross-reacts with dexamethasone (19.2%), but all other cross-reactivity values were less than 0.6%. To stabilize the variance, the base 10 logarithm of cortisol was used in data analysis.

Psychological Measures

We surveyed participants about their social media use, self-esteem, tendency to make social comparisons, and their ongoing stress, using validated and widely used assessments (Table 2). These factors were examined because they could alter the relationship between the physiological stress response and cell phone activities in this study. Assessments were self-reported with personal cell phones via Google Forms, following saliva sampling in the "Final" period (see Figure 1), and scale outcomes were calculated as defined by their developers. Scales are briefly described in Table 2, and additional details can be found in Appendix A.

Table 2. Validated psychological scales used in our study to quantify psychosocial factors. Additional details in Appendix A.

Psychological Scale	Measures:	Reference
Intensity of Social Media Use (Facebook Intensity Scale; Instagram Intensity Scale)	Participants' engagement with the platforms with respect to their number of "friends," daily time engagement, emotional investment, and extent to which one's daily activities are integrated with the platform	Ellison et al. 2007; Teo & Collinson 2019
Self-Esteem Scale	Feelings of self-worth and self-acceptance	Rosenberg 1965
Social Comparison Scale	Self-perception of social rank, relative attractiveness, and acceptance within one's social group, as pertains to social media	Allan & Gilbert 1995; Teo & Collinson 2019
Perceived Stress Scale	Self-assessed feelings of stress	Cohen et al. 1983

Statistical analysis

Using 2-group t-tests, we confirmed equality of the two groups (*Social Media then YouTube* and *YouTube then Social Media*) for age, psychological measures, and baseline measures of heart rate and cortisol. We confirmed our groups were similar in terms of sex and social media preference using chi-square tests. Because we randomly assigned individuals to group order, this assessment was necessary to ensure group equality on these key elements.

We confirmed a lack of carryover from the first experimental period to the second following standard protocol (Jones & Kenward 2015, Millikin & Johnson 2009). We fit a repeated measures model in a mixed model framework, incorporating fixed factors of treatment group (order), period, their interaction, and random effect of participant. Because of a strong period effect, we used the model to estimate pairwise comparisons and the treatment effect, adjusting for multiplicity using the false discovery rate (FDR) adjustment (Benjamani & Hochberg 1995). We then added each potential moderator (sex, age, and the four psychological measures) to the model to examine whether there was a change in the observed relationship between cell-phone treatment and physiological stress response; moderators were added independently and were not combined into a single model. We used self-reported sex and discretized age as under 25 years ("digital natives" who have grown up with social media) and 25 years or older. In the event of statistical significance in the 3-way interaction, we used model-based estimates of the outcomes at selected values of the moderator to better understand these interactions.

All statistical models used were evaluated for appropriateness with residual plots, including whether there were differences in mean response or variance among the study days. Statistical significance was defined as p (or adjusted p) ≤ 0.05. Linear modeling was completed with SAS 9.4 and all other descriptive statistics and plotting were completed in R version 4.0.2 (R Core Team 2020) using the *tidyverse* library (Wickham et al. 2019) in the RStudio IDE (RStudio Team 2020).

Results

We first verified that our two groups (Social Media then YouTube and YouTube then Social Media) were equivalent with respect to survey measures and demographics (Table 3). We note that Facebook Intensity was slightly higher in one group and was marginally statistically significant. Men and women were equally likely to use Facebook or Instagram (χ^2_1=0.240, p = 0.624), but on average those using Facebook were older than those using Instagram regardless of group ($T_{36.2}$=4.36, p<0.001; Facebook mean age 33.6 (2.5), Instagram mean age 21.5 (1.2)).

Table 3. Summary statistics of key demographics and test of equivalence. Value shown for each group is mean (standard error) N.

Variable	Social Media then YouTube	YouTube then Social Media	Test of group equivalence
Age	29.4 (2.3) 29	24.4 (1.9) 30	$T_{54.6}$ = 1.7, p = 0.092
Proportion Female	0.7 (0.1) 29	0.7 (0.1) 30	X^2_1 = 0.0, p = 0.928
Proportion Facebook Users	0.4 (0.1) 29	0.5 (0.1) 30	X^2_1 = 0.0, p = 0.883
Proportion Instagram Users	0.6 (0.1) 29	0.5 (0.1) 30	
Social Comparison	57.7 (2.1) 29	61.0 (2.4) 30	$T_{56.2}$ = -1.0, p = 0.310
Perceived Stress	39.9 (1.2) 29	39.2 (1.3) 30	$T_{56.8}$ = 0.4, p = 0.698
Self Esteem	21.1 (0.8) 29	20.3 (0.8) 30	$T_{57.0}$ = 0.7, p = 0.464
Social Media Intensity	24.7 (0.9) 29	23.1 (1.1) 30	$T_{55.9}$ = 1.1, p = 0.260
FB Intensity	26.5 (1.0) 12	22.7 (1.5) 14	$T_{21.6}$ = 2.1, p = 0.052
IG Intensity	23.4 (1.3) 17	23.4 (1.5) 16	$T_{30.1}$ = 0.0, p = 0.991

Carryover effects did not significantly differ for the two group orders for either heart rate ($F_{1,50}$ = 0.02, p = 0.878) or cortisol ($F_{1,51}$ = 1.21, p = 0.277). Heart rate and cortisol measures by experimental period are displayed in Figure 2. At baseline, heart rate was equivalent between groups (T_{51} = 0.59, p = 0.555) as was cortisol (T_{54} = 0.63, p = 0.53).

The period effect was statistically significant for both heart rate and cortisol in the linear models (Table 4), and the decrease in physiological response over the course of the study was not different between the two group orders (non-significant group order by period interaction, Table 4). Heart rate differed in all four periods (Figure 2), declining from Baseline to T1 and from Interim to T2, with a slight increase from T1 to Interim. This occurred regardless of group order. Heart rate during the Interim period was lower than during Baseline (Figure 2). From Baseline to T2, heart rate dropped an average of 7.3 (SE = 0.65) beats per minute. Cortisol concentrations were significantly lower following the second cell phone period (T2) than in the first two study periods (Figure 2). Participants' heart rates and cortisol levels ultimately decreased across the duration of the study, regardless of the cell phone treatments.

Considering the statistically significant period effect and non-significant interaction, we directly compared heart rate and cortisol responses between cell phone treatments during T1 only. There were

no significant differences in mean heart rate (T_{153} = -0.22, p = 0.827) or cortisol (T_{106} = 0.40, p = 0.687) between those viewing Social Media and YouTube during the T1 period.

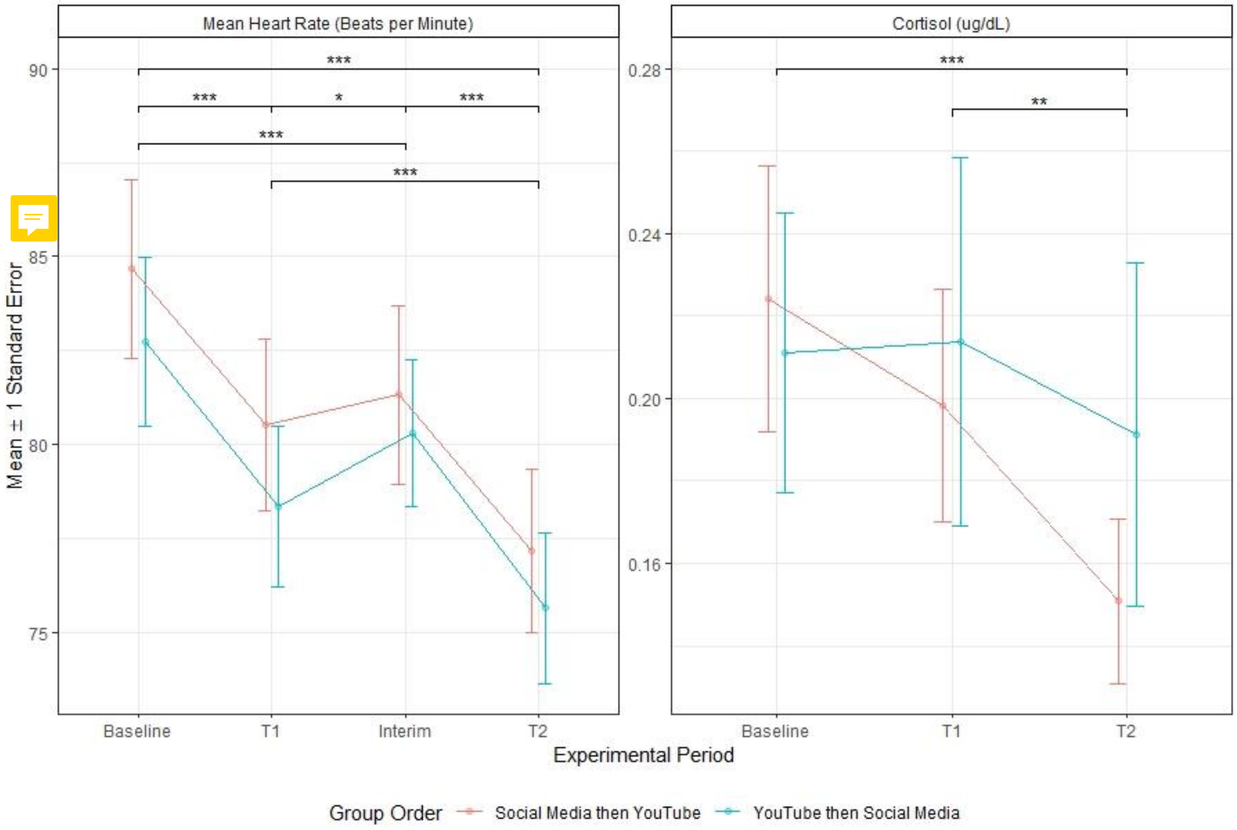

Figure 2. Summary of physiological measures by experimental period. Because there was no significant effect of Group Order or Group Order X time, comparisons of period are based on the main effect of period in the mixed model. Raw cortisol values are plotted; the inference is made from the mixed model using $\log_{10}$(Cortisol) values. Bars identify significantly different periods, *** indicates $p_{adj} < 0.001$, ** indicates $p_{adj} < 0.01$, and * indicates $p_{adj} < 0.05$. Adjustment using FDR (Benjamani & Hochberg 1995).

We separately added sex and age group to our models examining the effects of order and period and found no significant 3-way interaction with either factor; heart rate and cortisol responses were not moderated by either sex or age group. Next, we considered the moderating effects of social media platform (Facebook or Instagram) and psychological measures on heart rate and cortisol measurements. We found only a single significant 3-way interaction when examining the moderating effect of Intensity of Social Media use on cortisol ($F_{2,100}$ = 3.15, p = 0.047). High Intensity of Social Media use was associated with elevated cortisol at the baseline experimental period among those in the *Social Media then YouTube* group. As this only affects measurements before the experiment actually occurs, and doesn't reflect our experimental conditions, we do not believe it is relevant to our study conclusions and do not discuss it further.

Table 4. Analysis of variance results for study outcomes.

	Mean Heart Rate (BPM)				Cortisol (ug/dL)		
Base Model							
Fixed Effects	DF (num, den)	F-Value	p-Value		DF (num, den)	F-Value	p-Value
Group order	1, 51	0.3	0.587		1, 53.7	0.00	0.972
Experimental period	3, 153	62.54	<.0001		2, 105.9	9.56	<.001
Order*Period	3, 153	0.45	0.720		2, 105.9	2.17	0.119
Random Effects			Estimate				Estimate
Within participant			121.06				0.06
Residual			7.76				0.02

Data Availability Statement: Accession numbers and/or DOIs will be made available after acceptance.

Discussion

Because of concerns surrounding the pervasive use of social media, we set out to examine whether social media provokes a physiological stress response. The crossover design of our experiment allowed us to differentiate the effect of our social media treatment from the effect of treatment period, and we found a period effect but no treatment effect. Among those participating in our study, social media use did not elicit a physiological stress response and instead, the use of smartphones seemed to reduce activity of the sympathetic nervous system and HPA axis. Further, despite evidence of sex and age-specific differences in digital media use and psychological well-being (Twenge & Martin 2020), we found no evidence of age or sex modifying the physiological responses to our smartphone treatments. Additionally, neither psychological traits nor habits of social media use modified the physiological responses in our experiment. We were surprised by these findings because a growing body of literature suggests that excessive smartphone use, and social media use in particular, is detrimental to mental and physical health (e.g. Anderson et al. 2008, Twenge et al. 2018, Woods and Scoot 2016). We expected to find that social media use would trigger a measurable physiological stress response and that this might help explain its contribution to psychiatric and physical morbidities.

Despite the absence of a physiological stress response to our social media treatment, our findings do not preclude the possibility that social media use induces psychological stress. Among studies examining the physiological response to psychological stressors, some have elicited robust physiological responses (e.g. Bae et al. 2019, Dickerson & Kemney 2004, Woody et al. 2018), while other studies fail to find an association (Leis & Lautenbach 2020, Lupis et al. 2014). These contradictory findings may be explained by variations in the cognitive appraisal of a stressor; some perceptions of stress are more likely to elicit a physiological response than others (Denson et al. 2009; Dickerson & Kemeny 2004, Tomaka et al. 1993). Specifically, stressors that are appraised as *uncontrollable*, *novel*, *challenging*, and/or *threatening* are most likely to activate the HPA axis and sympathetic division of the autonomic nervous system (Denson et al. 2009). When stressors are appraised in this way, the situation may be perceived as requiring extra resources and thus physiological and behavioral modification are initiated to help provide these

resources. In reference to the findings in our study, content viewed on smartphones may not have been appraised as uncontrollable, novel, challenging, or threatening and thus did not provoke a physiological stress response.

Mobile devices are *controllable* and are not *novel*. While cell phone alerts solicit attention, users can control the amount and type of information disseminated in their feeds. Further, cell phones have lost their novelty and are ubiquitous in our culture. Perhaps when cell phones were novel their use prompted a physiological response, but more recently this response has become habituated, such that a diminished physiological response occurs with repeated exposure to the same stimulus (Costoli et al. 2004, Grissom & Bhatnager 2009). Our participants may have been habituated individuals using their cell phones in the manner to they have become accustomed. Consequently, a physiological stress response was not provoked by our experimental treatment.

When we gave our study instructions, fitted heart rate monitors, and collected saliva samples, we may have inadvertently introduced a sense of novelty and uncontrollability, which has been shown to elicit a physiological stress response (Salvador et al. 2003, van Paridon et al. 2017, Peters et al. 1998). This could explain the relatively higher heart rate during the Baseline and Interim study periods than during the cell phone treatment periods; the sympathetic nervous system seemed to be more active during periods of smartphone abstinence than during periods of smartphone use. Similar findings have been reported by Johnshoy et al. (2020) and Rus and Tiemensma (2018) who found that social media use on personal devices attenuated the physiological stress response to an acute stressor (Trier Social Stress Test). It may be that contact with smartphones offers a form of social support, even if an individual does not interact with members of one's social group through social media. Access to the phone itself may be a reassuring comfort in times of uncertainty.

Social media and YouTube may not have been perceived as *challenging* or *threatening*. While we did not directly survey feelings of challenge or threat, our social comparison and self-esteem scales offered insight into such emotions. Those who consistently rank themselves as inferior on the social comparison scale may have felt threatened by online interactions with their "friends," and those who ranked themselves as superior might have felt challenged. Similarly, it can be assumed that those with low self-esteem may have felt more challenged or threatened by social media than those with higher self-esteem. Studies have suggested that threats to the social self (i.e. situations that could lead to rejection of an individual's self-worth) often result in psychobiological responses, including HPA axis and cardiovascular activation (Dickerson and Kemeny 2004, Woody et al. 2018). As such, we expected to find a heightened physiological stress response when individuals in our study viewed social media. However, we found no relationships between physiological measures of stress and the responses to self-esteem or social comparison scales.

Psychological stressors are first processed by higher brain centers before activating physiological responses (Kudielka and Kirschbaum 2005). In our study it seems likely that this higher-order processing resulted in the social media and YouTube content being appraised as benign; posts that could be construed as either threatening or challenging may have undergone refinement by cognitive processes so that they were appraised as harmless. It may not be social media, per se, that evokes a response but rather the perception of social media content that determines the physiological response. Further, social media users may have "pruned their feed" such that threatening or challenging posts were entirely eliminated from view.

Limitations

As our study population reflected a subpopulation of individuals who consented to engage with their social media accounts and smartphones for an hour, this group may not be representative of the general population. Also, we did not control the social media content viewed by any of the participants. While we maintain that our study adequately assessed responses to participants' *typical use* of social media, we acknowledge that our design, which allowed participants to view their preferred content, may have introduced some uncontrolled variability to our study.

Our study does not preclude the possibility that social media use can be harmful. Several studies have suggested that social media use can promote negative emotions and distress (e.g. Tobin et al. 2015, Verduyn et al. 2015, Wenninger et al. 2014), and digital technologies (including social media) have been shown to displace beneficial activities (Przybylski & Weinstein 2017), such as school work (Neuman 1988) and exercise (Anderson et al. 2008). We did not measure such potential negative outcomes. In light of the current online environment, in which divisive discourse and extremist views are commonplace, it may be important to examine the emotional and behavioral responses to social media activities even if they do not increase heart rate and cortisol levels.

Conclusions

While it may be prudent to consider the implications of unconstrained social media, the dangers of new technologies are often overblown. In the Victorian age, print publications were thought to distort human learning and communications, and the wireless telegraph was feared to isolate users from human interactions (Dickson 2016). The results of our study add to a small body of evidence suggesting that social media use may in fact serve as a stress coping tool (e.g. Johnshoy et al. 2020, Rus & Tiemensma 2018). We found that social media use does not provoke a physiological stress response but instead that sitting still and scrolling through content on smart phones likely activates the parasympathetic nervous system and depresses activity of the HPA axis.

Acknowledgements

This study was conducted as part of a summer research experience for undergraduates who participated in the INBRE Summer Scholars program at College of Western Idaho. We are indebted to the following students for their help with and dedication to this project: Brittany Beers, Zoey Carr, Elizabeth Carter, Cristiana Holmes, Abdi Mohamed, Brandi Sweet, Riley Woodworth.

Special thanks to Michelle Fellows for her support in early concept development and Devaleena Pradhan and Keith Sockman for helpful comments on the manuscript.

Literature Cited

Afifi, T.D., Zamanzadeh, N., Harrison, K., & Callejas, M.A. (2018). WIRED: The impact of media and technology use on stress (cortisol) and inflammation (interleukin IL-6) in fast paced families. Computers in Human Behavior, 81, 265–273. https://doi.org/10.1016/j.chb.2017.12.010.

Alfano, C.A., Zakem, A.H., Costa, N.M., Taylor, L. K., & Weems, C. F. (2009). Sleep problems and their relation to cognitive factors, anxiety, and depressive symptoms in children and adolescents. Depression and Anxiety, 26(6), 503-12. https://doi.org/10.1002/da.20443

Allan, S. & Gilbert, P. (1995). A social comparison scale: Psychometric properties and relationship to psychopathology. Personality and Individual Differences, 19(3), 293–299. https://doi.org/10.1016/0191-8869(95)00086-L

American Academy of Pediatrics council on communications and media. 2016. Media Use in School-Aged Children and Adolescents. Pediatrics, 138(5).

Anderson, S.E., Economos, C.D., & Must, A. (2008). Active play and screen time in US children aged 4 to 11 years in relation to sociodemographic and weight status characteristics: A nationally representative cross-sectional analysis. BMC Public Health, 8(1), 366. https://doi.org/10.1186/1471-2458-8-366

Bae, Y.J., Reinelt, J., Netto, J., Uhlig, M., Willenberg, A., Ceglarek, U., Villringer, A., Thiery, J., Gaebler, M., & Kratzsch, J. (2019). Salivary cortisone, as a biomarker for psychosocial stress, is associated with state anxiety and heart rate. Psychoneuroendocrinology, 101, 35–41. https://doi.org/10.1016/j.psyneuen.2018.10.015

Barry, C.T., Sidoti, C.L., Briggs, S., Reiter, S., & Lindsey, R.A. (2017). Adolescent social media use and mental health from adolescent and parent perspectives. Journal of Adolescence, 61, 1–11. https://doi.org/10.1016/j.adolescence.2017.08.005

Baumeister, R.F. & Leary, M.R. (1995). The need to belong: Desire for interpersonal attachments as a fundamental human motivation. Psychological Bulletin, 117(3), 497–529. https://doi.org/10.1037/0033-2909.117.3.497.

Becker, L., & Rohleder, N. (2019). Time course of the physiological stress response to an acute stressor and its associations with the primacy and recency effect of the serial position curve. PLOS ONE, 14(5), e0213883. https://doi.org/10.1371/journal.pone.0213883

Benjamini, Y. & Hochberg, Y. 1995. Controlling the false discovery rate - a practical and powerful approach to multiple testing. Journal of the Royal Statistical Society Series B-Statistical Methodology, 57(1), 289-300. https://doi.org/10.1111/j.2517-6161.1995.tb02031.x

Bhagwagar, Z., Hafizi, S., & Cowen, P.J. (2005). Increased salivary cortisol after waking in depression. Psychopharmacology, 182(1), 54–57. https://doi.org/10.1007/s00213-005-0062-z

Cohen, S., Kamarck, T., & Mermelstein, R. (1983). A global measure of perceived stress. Journal of Health and Social Behavior, 24(4), 385. https://doi.org/10.2307/2136404

Costoli, T., Bartolomucci, A., Graiani, G., Stilli, D., Laviola, G., & Sgoifo, A. (2004). Effects of chronic psychosocial stress on cardiac autonomic responsiveness and myocardial structure in mice. American Journal of Physiology - Heart and Circulatory Physiology, 286(6), 2133–2140. https://doi.org/10.1152/ajpheart.00869.2003

Dempsey, A.E., O'Brien, K.D., Tiamiyu, M.F., & Elhai, J.D. (2019). Fear of missing out (FoMO) and rumination mediate relations between social anxiety and problematic Facebook use. Addictive Behaviors Reports, 9, 100150–100150. https://doi.org/10.1016/j.abrep.2018.100150

Denson, T.F., Spanovic, M., & Miller, N. (2009). Cognitive appraisals and emotions predict cortisol and immune responses: A meta-analysis of acute laboratory social stressors and emotion inductions. Psychological Bulletin, 135(6), 823–853. https://doi.org/10.1037/a0016909

Dickerson, S.S. & Kemeny, M.E. (2004). Acute stressors and cortisol responses: A theoretical integration and synthesis of laboratory research. Psychological Bulletin, 130(3), 355–391. https://doi.org/10.1037/0033-2909.130.3.355

Dickson, M. "The Victorians had the same concerns about technology as we do." The Conversation, http://theconversation.com/the-victorians-had-the-same-concerns-about-technology-as-we-do-60476. Accessed 8 Sept. 2021.

Diener, E., Suh, E.M., Lucas, R.E., & Smith, H.L. (1999). Subjective well-being: Three decades of progress. Psychological Bulletin, 125(2), 276–302. https://doi.org/10.1037/0033-2909.125.2.276.

Ehrenreich, S.E. & Underwood, M.K. (2016). Adolescents' internalizing symptoms as predischoctors of the content of their Facebook communication and responses received from peers. Translational Issues in Psychological Science, 2(3), 227–237. https://doi.org/10.1037/tps0000077.

Ellison, N.B., Steinfield, C., & Lampe, C. (2007). The benefits of Facebook "friends:" Social capital and college students' use of online social network sites. Journal of Computer-Mediated Communication, 12(4), 1143–1168. https://doi.org/10.1111/j.1083-6101.2007.00367.x

Engert, V., Vogel, S., Efanov, S. I., Duchesne, A., Corbo, V., Ali, N., & Pruessner, J. C. (2011). Investigation into the cross-correlation of salivary cortisol and alpha-amylase responses to psychological stress. Psychoneuroendocrinology, 36(9), 1294–1302. https://doi.org/10.1016/j.psyneuen.2011.02.018

Fredriksen, K., Rhodes, J., Reddy, R. & Way, N. (2004). Sleepless in Chicago: Tracking the effects of adolescent sleep loss during the middle school years. Child Development, 75(1), 84- 95. https://www.jstor.org/stable/3696567

Great Britain. Parliament. House of Commons. Select Committee on Science Technology, issuing body. (2019). Impact of social media and screen-use on young people's health (HC (Series) (Great Britain. Parliament. House of Commons); 822 2017-19). London.

Grissom, N. & Bhatnagar, S. (2009). Habituation to repeated stress: Get used to it. Neurobiology of Learning and Memory, 92(2), 215–224. https://doi.org/10.1016/j.nlm.2008.07.001

Hawes, T., Zimmer-Gembeck, M.J., & Campbell, S.M. (2020). Unique associations of social media use and online appearance preoccupation with depression, anxiety, and appearance rejection sensitivity. Body Image, 33, 66–76. https://doi.org/10.1016/j.bodyim.2020.02.010

Jezova, D., Makatsori, A., Duncko, R., Moncek, F., & Jakubek, M. (2004). High trait anxiety in healthy subjects is associated with low neuroendocrine activity during psychosocial stress. Progress in Neuro-Psychopharmacology and Biological Psychiatry, 28(8), 1331–1336. https://doi.org/10.1016/j.pnpbp.2004.08.005

Johnshoy, Q., Moroze, E., Kaser, I., Tanabe, A., Adkisson, C., Hutzley, S., Cole, C., Garces, S., Stewart, K., & Campisi, J. (2020). Social media use following exposure to an acute stressor facilitates recovery from the stress response. Physiology & Behavior, 223, 113012–113012. https://doi.org/10.1016/j.physbeh.2020.113012

Jones, B. & Kenward, M.G. (2015). Design and analysis of cross-over trials. 3rd Edition. Monographs on Statistics and Applied Probability 138. CRC Press: Boca Raton, FL.

Juster, R-P., McEwen B.S., & Lupien, S.J. (2010). Allostatic load biomarkers of chronic stress and impact on health and cognition. Neuroscience and Biobehavioral Reviews, 35, 2-16.

Kelly, Y., Zilanawala, A., Booker, C., & Sacker, A. (2018). Social media use and adolescent mental health: Findings from the UK millennium cohort study. EClinicalMedicine, 6, 59–68. https://doi.org/10.1016/j.eclinm.2018.12.005

Kudielka, B. M., Schommer, N. C., Hellhammer, D. H., & Kirschbaum, C. (2004). Acute HPA axis responses, heart rate, and mood changes to psychosocial stress (TSST) in humans at different times of day. Psychoneuroendocrinology, 29(8), 983–992. https://doi.org/10.1016/j.psyneuen.2003.08.009

Kudielka, B. M. & Kirschbaum, C. (2005). Sex differences in HPA axis responses to stress: A review. Biological Psychology, 69, 113–132. https://doi.org/10.1016/j.biopsycho.2004.11.009

Leis, O. & Lautenbach, F. (2020). Psychological and physiological stress in non-competitive and competitive esports settings: A systematic review. Psychology of Sport and Exercise, 51, 101738. https://doi.org/10.1016/j.psychsport.2020.101738

Lin, L.Y., Sidani, J.E., Shensa, A., Radovic, A., Miller, E., Colditz, J.B., Hoffman, B.L., Giles, L.M., & Primack, B.A. (2016). Association between social media use and depression among U.S. young adults. Depression and Anxiety, 33, 323-331.

Lupis, S.B., Lerman, M., & Wolf, J.M. (2014). Anger responses to psychosocial stress predict heart rate and cortisol stress responses in men but not women. Psychoneuroendocrinology, 49(1), 84–95. https://doi.org/10.1016/j.psyneuen.2014.07.004

McEwen, B.S. (1998). Protective and damaging effects of stress mediators. New England Journal of Medicine, 338(3), 171–179. https://doi.org/10.1056/NEJM199801153380307

Milliken, G.A. & Johnson, D.E. (2009). Analysis of messy data volume 1. Designed experiments. 2nd Edition. Chapman and Hall / CRC: Boca Raton, FL.

Morin-Major, J.K., Marin, M.F., Durand, N., Wan, N., Juster, R.P., & Lupien, S.J. (2016). Facebook behaviors associated with diurnal cortisol in adolescents: Is befriending stressful? Psychoneuroendocrinology, 63, 238–246. https://doi.org/10.1016/j.psyneuen.2015.10.005

Neuman, S.B. (1988). The displacement effect: Assessing the relation between television viewing and reading performance. Reading Research Quarterly, 23(4), 414. https://doi.org/10.2307/747641

Owens, A. P., Low, D.A., Iodice, V., Mathias, C.J., Critchley, H.D. (2017). Emotion and the autonomic nervous system - a two-way street: insights from affective, autonomic and dissociative disorders. In: Stein, John (ed.) Reference module in neuroscience and biobehavioral psychology. Elsevier, pp. 1-15. ISBN 9780128093245.

Peters, M.L., Godaert, G.L.R., Ballieux, R.E., Van Vliet, M., Willemsen, J.J., Sweep, F.C.G.J., & Heijnen, C.J. (1998). Cardiovascular and endocrine responses to experimental stress: Effects of mental effort and controllability. Psychoneuroendocrinology, 23(1), 1–17. https://doi.org/10.1016/S0306-4530(97)00082-6

Pew Research Center, Washington, D.C. (2018). Social Media Use in 2018. https://www.pewresearch.org/internet/2018/03/01/social-media-use-in-2018/

Przybylski, A.K., Murayama, K., DeHaan C.R., & Gladwell V. (2013). Motivational, emotional, and behavioral correlates of fear of missing out. Computers in Human Behavior, 29(4), 1841-1848. https://doi.org/10.1016/j.chb.2013.02.014

Przybylski, A.K. & Weinstein, N. (2017). A large-scale test of the goldilocks hypothesis: Quantifying the relations between digital-screen use and the mental well-being of adolescents. Psychological Science, 28(2), 204–215. https://doi.org/10.1177/0956797616678438

R Core Team (2020). R: A language and environment for statistical computing. R Foundation for Statistical Computing, Vienna, Austria. URL https://www.R-project.org/.

Rauch, S.M., Strobel, C., Bella, M., Odachowski, Z., & Bloom, C. (2014). Face to face versus Facebook: Does exposure to social networking web sites augment or attenuate physiological arousal among the socially anxious? Cyberpsychology, Behavior, and Social Networking, 17(3), 187–190. https://doi.org/10.1089/cyber.2012.0498

Rosenberg, M. (1965). Society and the adolescent self-image. Princeton, NJ: University Press

Rosmond, R. (2003). Stress induced disturbances of the HPA axis: A pathway to Type 2 diabetes? Med Sci Monit, 9(2), 35-39.

RStudio Team (2020). RStudio: Integrated Development for R. RStudio, PBC, Boston, MA URL http://www.rstudio.com/.

Rus, H.M. & Tiemensma, J. (2017). Social media under the skin: Facebook use after acute stress impairs cortisol recovery. Frontiers in Psychology, 8, 1–10. https://doi.org/10.3389/fpsyg.2017.01609.

Rus, H.M. & Tiemensma, J. (2018). Social media as a shield: Facebook buffers acute stress. Physiology and Behavior, 185, 46–54. https://doi.org/10.1016/j.physbeh.2017.12.021

Salimetrics (2015). Expanded Range High Sensitivity Salivary Cortisol Enzyme Immunoassay Kit. State College, PA.

Salvador, A., Suay, F., González-Bono, E., & Serrano, M.A. (2003). Anticipatory cortisol, testosterone and psychological responses to judo competition in young men. Psychoneuroendocrinology, 28(3), 364–375. https://doi.org/10.1016/S0306-4530(02)00028-8

Sapolsky, R.M. (2004). Social status and health in humans and other animals. Annual Review of Anthropology, 33(1), 393–418. https://doi.org/10.1146/annurev.anthro.33.070203.144000

Shakya, H.B. & Christakis, N.A. (2017). Association of Facebook use with compromised well-being: a longitudinal study. American Journal of Epidemiology, 185(3), 203–11. https://doi.org/10.1093/aje/kww189.

Shafi, R.M.A., Nakonezny, P.A., Miler, K.A., Desai, J., Almorsy A.G., Ligezka, A.N., Morath, B.A., Romanowica, M., and Croarkin, P.E. (2021). An exploratory study of clinical and physiological correlates of problematic social media use in adolescents. Psychiatry Research, 302, 114020. https://doi.org/10.1016/j.psychres.2021.114020

Teo, N.S.Y. & Collinson, S.L. (2019). Instagram and risk of rumination and eating disorders: An Asian perspective. Psychology of Popular Media Culture, 8(4), 491-508. https://doi.org/10.1037/ppm0000205

Tobin, S.J., Vanman, E.J., Verreynne, M., & Saeri, A.K. (2015). Threats to belonging on Facebook: Lurking and ostracism. Social Influence, 10(1), 31–42. https://doi.org/10.1080/15534510.2014.893924

Tomaka, J., Blascovich, J., Kelsey, R.M., & Leitten, C.L. (1993). Subjective, physiological, and behavioral effects of threat and challenge appraisal. Journal of Personality and Social Psychology, 65(2), 248–260. https://doi.org/10.1037/0022-3514.65.2.248

Twenge, J.M. (2017). Have smartphones destroyed a generation? The Atlantic. September 2017 issue. Accessed online at: https://www.theatlantic.com/magaz ine/archive/2017/09/has-the-smartphone-destroyed-a-generation/534198

Twenge, J.M. & Martin, G.N. (2020). Gender differences in associations between digital media use and psychological well-being: Evidence from three large datasets. Journal of Adolescence, 79, 91–102. https://doi.org/10.1016/j.adolescence.2019.12.018

Twenge, J.M., Martin, G.N. & Campbell, W.K. (2018). Decreases in psychological well-being among american adolescents after 2012 and links to screen time during the rise of smartphone technology. Emotion, 18(6), 765–80. https://doi.org/10.1037/emo0000403.supp.

van Paridon, K.N., Timmis, M.A., Nevison, C.M., & Bristow, M. (2017). The anticipatory stress response to sport competition; A systematic review with meta-analysis of cortisol reactivity. BMJ Open Sport Exerc. Med., 3(1), e000261. https://doi.org/10.1136/bmjsem-2017-000261

Verduyn, P, Lee, D.S., Park, J., Shablack, H., Orvell, A., Bayer, J., Ybarra, O., Jonides, J., & Kross, E. (2015). Passive Facebook usage undermines affective well-being: Experimental and longitudinal evidence. J Exp Psychol Gen., 144, 480-488. https://doi.org/10.1037/xge0000057

Verduyn, P., Ybarra, O., Résibois, M., Jonides, J., & Kross, E. (2017). Do social network sites enhance or undermine subjective well-being? A critical review. Social Issues and Policy Review, 11(1), 274–302. https://doi.org/10.1111/sipr.12033

Wang, P., Xie, X., Wang, X., Wang, X., Zhao, F., Chu, X., Nie, J., & Lei, L. (2018). The need to belong and adolescent authentic self-presentation on SNSs: A moderated mediation model Involving FoMO and perceived social support. Personality and Individual Differences, 128, 133–38. https://doi.org/10.1016/j.paid.2018.02.035.

Wenninger, H., Krasnova, H., & Buxmann, P. 2014. Activity matters: Investigating the influence of Facebook on life satisfaction of teenage users. Proc. European Conf. on Information Systems (ECIS) 2014, Tel Aviv, Israel.

Wickham, H., Averick, M , Bryan, J. , Chang, W., McGowan, L.D. , François, R., Grolemund, G., Hayes, A., Henry, L., Hester, J., Kuhn, M., Pedersen, T.L., Miller, E., Bache, S.M., Müller, K., Ooms, J., Robinson, D., Seidel, D.P., Spinu, V., Takahashi, K., Vaughan, D., Wilke, C., Woo, K., & Yutani, H. (2019). Welcome to the tidyverse. Journal of Open Source Software, 4(43), 1686, https://doi.org/10.21105/joss.01686

Woods, H.C. & Scott, H. 2016. #Sleepyteens: Social media use in adolescence is associated with poor sleep quality, anxiety, depression and low self-esteem. Journal of Adolescence, 51, 41–49. https://doi.org/10.1016/j.adolescence.2016.05.008.

Woody, A., Hooker, E.D., Zoccola, P.M., & Dickerson, S.S. (2018). Social-evaluative threat, cognitive load, and the cortisol and cardiovascular stress response. Psychoneuroendocrinology, 97, 149–155. https://doi.org/10.1016/j.psyneuen.2018.07.009

Appendix A.

Psychological Scales

Intensity of Social Media Use

Our participants selected either the Facebook or Instagram Intensity Scale to measure the degree to which they engage with their chosen social media platform. The scales for each platform are approximately equivalent; the language in the Facebook Intensity Scale, created by Ellison et al. (2007), was minimally modified by Teo and Collinson (2019) to apply to Instagram as the Instagram Intensity Scale. The Facebook and Instagram intensity scales measure participants' engagement with these platforms with respect to their number of "friends," daily time engagement, emotional investment, and extent to which one's daily activities are integrated with the platform. The Facebook and Instagram Intensity Scales consist of six statements in which participants choose an answer ranging from 1) *strongly disagree* to 5) *strongly agree.* There are three open-ended questions regarding the number of social media friends and followers and the frequency with which they checked their social media feeds within the past week. Responses to these three questions are collapsed into an ordinal scale that also ranges from 1 to 5. Responses on this scale were summed for each participant, with higher scores

indicating greater intensity of social media use. One participant left two items blank on the Facebook Intensity Scale; these two items were imputed with the overall mean for these items.

Self-Esteem Scale

Self-esteem was measured with the Rosenberg Self-Esteem Scale (Rosenberg 1965) which quantifies feelings of self-worth and self-acceptance. The scale contains 10 statements about feelings and attitudes towards one's self. Responses to statements are made on a four-point scale anchored by *strongly agree* and *strongly disagree*. After accounting for items with reversed scoring, a summed self-esteem score was calculated for each participant. Higher numbers indicate greater self-esteem. While subscales are considered reliable and valid for this instrument, we used the overall score. One participant neglected a single item on the Self-Esteem Scale, and this was imputed using the participant's response to the other item on the same subscale.

Social Comparison Scale

The Social Comparison Scale (Allan & Gilbert 1995) assesses self-perception of social rank, relative attractiveness, and acceptance within one's social group. The scale used in our study was modified by Teo & Collinson (2019) to pertain specifically to social media. This scale consists of 10 incomplete sentences in which participants select a relative number between two bipolar constructs representing their self-perception. For instance, a participant using Facebook would select a number between 1 and 10 in response to the statement "When I compare myself to others on Facebook, I feel *Inferior* 1 2 3 4 5 6 7 8 9 10 *Superior*." A sum of quantified responses is calculated as the measure outcome, with higher scores indicating that the participant perceives a more favorable social position for themselves, compared to others within their social group.

Perceived Stress Scale

Participants' self-assessment of stress was measured with The Perceived Stress Scale (Cohen et al. 1983). This scale consists of 14 questions pertaining to the participants' thoughts and feelings during the previous month. Response options are made on a four-point scale ranging from never to very often. After accounting for items with reversed scoring, a sum of scores was calculated for each participant, with higher scores indicating higher stress. To avoid the possibility that thoughts of stress prompted by this scale could influence responses on other scales, this was the last scale administered in our study.
