# Supplementary material for: Social media does not elicit a physiological stress response as measured by heart rate and salivary cortisol over 20-minute sessions of cell phone use
Source: PLoS One. 2024 Apr 3;19(4):e0298553. doi: 10.1371/journal.pone.0298553 (PMC10990243; doi:10.1371/journal.pone.0298553)
Supplement: Attachment — Submitted filename: PONE-D-23-15507_R2_R2.pdf [file pone.0298553.s006.pdf]

Page 7. Comment number 7: For the sake of clarity, the authors propose the use of "short-term" instead of the intervention time (20 min) used in the study. The new title proposed is not acceptable for this reviewer, unless further evidence is provided showing that the typical use of social media in the population studied is close to their intervention time. Otherwise, it is strongly recommended by this reviewer to find a more accurate title, for the sake of good practice.

Page 8. Comment number 2: Only 1 out of the 6 studies presented in the table did not report a difference in cortisol and social media usage. The following 4 investigations have shown results in the same direction: cortisol levels and social media usage are positively associated. (Morin-Major et al. (2016): the higher the diurnal cortisol level the higher the number of Facebook friends; Rus & Tiemensma (2017): the higher the cortisol levels, the higher the social media use; Afifi et al. (2018): the greater the cortisol awakening response, the more frequent use of social media; and Shafi et al. (2021): the higher the cortisol levels, the more usage of social media). For this reviewer, 1 study out of 6 is not enough evidence to claim that findings are inconsistent, when 4 out of 6 have shown the same direction of response. Please, either provide more references showing no differences and/or negative associations between cortisol levels and social media usage or avoid using inconsistent results as an argument here.

Page 9. Comment number 3: Assumptions are made as part of the introduction. Lack of control of potential factors that could influence one of the variables investigated should be mentioned in the section about potential limitations of the study. This reviewer disagrees with the assumption made by the authors that the effects will last for the entire duration of the study. As an example of that: participant A took a coffee at 6 AM; participant B took a coffee at 11:30 AM. This issue is not being solved by using a repeated measures design, as mentioned by the authors in their response. By the time the participants were tested (12:30 PM) the effect of caffeine on their experiment is clearly different from participant A (very likely no effect) to participant B (very likely to have an ergogenic effect). We could hypothesize that participant A and B took their coffees at the same time of the day every day, so if they were both involved in two separate testing sessions, we could agree that caffeine is very likely to have the same effect during both experimental days. However, the investigation presented by the authors uses a single-day design. Hence why this reviewer is making a big emphasis on the importance of potential factors that may have an influence or their results. It is understandable that the authors may have not recognized this point when designing the study. But it is expected, at least, a mention in the potential limitations of their experiment.

Page 9. Comment number 7: if possible, add standard deviation and the range of the start of the testing. It is good practice to give the reader an idea of the accuracy of the starting time, that usually might be delayed due to logistical issues. Also, it would give additional information regarding potential outliers due to a very late start, compared to the average. However, this reviewer understands that this could not be taken into consideration when collecting the data.

Page 14. Comment number 1: It is good practice to describe in the corresponding section about statistics the p value set up for significance. In most studies, defined as a value smaller than 0.05. Removing this from your manuscript is not acceptable for this reviewer.
