# Supplementary material for: Social media does not elicit a physiological stress response as measured by heart rate and salivary cortisol over 20-minute sessions of cell phone use
Source: PLoS One. 2024 Apr 3;19(4):e0298553. doi: 10.1371/journal.pone.0298553 (PMC10990243; doi:10.1371/journal.pone.0298553)
Supplement: Attachment — Submitted filename: Response to Reviewers.docx [file pone.0298553.s005.docx]

5 September, 2023

Dear Dr. Castillo-Navarrete,

We appreciate the opportunity to resubmit our revised manuscript. We believe that a strength of our study was the cross-over design to evaluate our treatment conditions, but we also recognize the complexity of this design. We have re-written paragraphs in response to both reviewers’ comments which indicate where we fell short in our initial explanation. We are hopeful that the reviewers will find our second attempt not only addresses their concerns but also clarifies the process for all readers.

To address each of the reviewers’ comments found in the Editorial Manager document, we have included a table which pairs each comment with our response. In addition, we have responded below (in red) to comments found in “Review Comments to Author.” Some of these Review Comments are duplicates of those found in the Editorial Manager document; we have responded here, but additional responses may be found in the table below. Please note that the page references on the attached table refer to those in the Editorial Manager documents containing reviewers’ comments (not to those of our manuscript submission).

We recognize that our manuscript is not fully formatted to the specifications for PLOS ONE, and we will respond promptly to a provisional Editorial Accept decision for the remainder of the changes not implemented here (e.g., graphics, reference brackets). We have left the Appendix as part of the manuscript, but include a note (to ourselves) to submit as a separate file with our final submission. We noticed that Fig. 2 referred to “experimental period,” but that Fig. 1 referred to “study period.” We standardized our language, and both now refers to “study period.”

Again, we thank you for the opportunity to publish in PLOS ONE and for the time and attention that the reviewers provided. We look forward to hearing from you again soon.

Sincerely,

Suzanne Oppenheimer

Review Comments to the Author

**Reviewer #1**: 
1. Terminology and writing: While the study appears sound, the language is unclear, potentially failing to communicate the message. Specifically:

- The authors use the verb "provoke" in the title, abstract, introduction, discussion and conclusions. It appears that the use of the verb "provoke" is inconsistent with the literature that has been introduced (page 1, the articles cited refer to "rising, augmenting and delaying" stress levels, not "causing" as the verb "provoke" suggests) and with the aim of the study (page 2, "Our study directly evaluates the relationship between…").
We understand that this word can have multiple meanings and interpretations. On page 1, we have changed the word “provoke” to “influence” and to “elicit” in the title of the manuscript. In other instances, our intended meaning of “provoke” is “causing,” and we have not made changes to the wording in these locations.

- In the introduction (page 1), the term "curated" does not align with the meaning intended by the authors (WordReference: From the verb curate. 1. a member of the clergy employed to assist a rector or vicar.)

We have not used the noun “curate” but instead the verb "curated” to mean *carefully chosen* (<https://www.merriam-webster.com/dictionary/curated>) which is aligned with our YouTube selection and chose not to change the article text.

- The term "routine use" (title and introduction, page 2) may be misused. Although the word "routine" may suggest that the intervention is performed regularly, it is important to note that it is only assessed once in this study. The authors asked participants to use social media as "typical", but the study design was not longitudinal.

We agree with the reviewer and believe our own biases affected our wording choice. Our aim was to encourage users to act, during the study periods, in a “routine manner”, but we agree that the usage of the word “routine” in this context is incorrect. In the introduction as well as throughout the manuscript, we have changed the word “routine” to more carefully reflect our intended meaning.

- The experiment implementation is described clearly (1 group per day: 15 people on the 1st, 21 on the 2nd, 23 on the 3rd). However, it also says, "On a given day, all participated simultaneously".

We initially hoped that this streamlined wording was understandable but clearly, it was not. We meant, on (say) the first day, that the 15 participants participated at the same time. We hope that by adding “On each day” at the beginning of this sentence, as described in response to this concern in the table for Reviewer 1, better reflects our true meaning.

-The term "period effect" used by the authors is unclear and requires further explanations. Both experimental conditions had the same duration (page 4). On page 7, the authors write "the course of the study". The results (page 8) report the "period effect", but the sentence is complex and lengthy. It would be beneficial to include a clear explanation earlier in the manuscript rather than at the end of the Results section (page 8, "Participants' heart rates and cortisol levels ultimately decreased across the duration of the study, regardless of the cell phone treatments.").

We have added a more detailed description of the study design and defined the period effect, treatment order, treatment effect, and carryover effects with respect to this study, as well as added references where these designs have been used in other fields. With this more detailed explanation, we believe the entire statistical analysis and results section will become clearer to the reviewers as well as our readers. We hope that our renewed attempt to explain the cross-over study design will enhance the manuscript and improve our readers’ understanding of the design, results, and analyses. The period effect explicitly addresses whether results from the first 20-minute experimental period, regardless of which treatment was received, is different from the second 20-minute experimental period, again regardless of treatment received. With this rewritten text, we are hopeful that the reviewer will find the “period effect” concept, central to our conclusions, is much clearer..

- Page 9 reports "no significant differences in mean heart rate (T153 = -0.22, p = 0.827) or cortisol (T106 = 0.40, p = 0.687) between those viewing Social Media and YouTube during the T1 period.". The sentence contradicts the previously mentioned results.

Please see our response to the prior concern. Because of the statistically significant period effect, we analyzed the treatment effect (whether heart rate or cortisol differed depending on whether a participant viewed Social Media or YouTube) using a simple two-group t-test. We hope that the new explanation in the manuscript clarifies that there is in fact no contradiction.

2. Organisation of the report content: the authors should reorganise the following section to avoid confusion while reading and understanding how the study was implemented.

- In the abstract, essential methodological choices are left out.

We have modified the abstract following reviewers’ suggestions.

- The study rationale is well-explained by the literature background. However, the study hypothesis and expectations are unclear (page 2). Despite it, the aim of the experiment is clearly stated.

Our hypothesis statement can be found in the last sentence of the introduction.

- The ethics are in line with the expected standards. However, the participants' paragraph also describes info that should be part of the experiment procedure (page 3).

We have changed the subheadings, such that information regarding Participants & Overall procedures fall under the same subheading. Otherwise, we would necessarily need to repeat information that is relevant to both sections.

-The sentence on page 3, "We employed a two-period crossover design (Jones & Kenward 2015, Millikin & Johnson 2009) to evaluate the physiological response to social media relative to non-evocative content on YouTube." It should be better explained.

We have revised our description of the experimental method and elements of the crossover design. Please see our responses to specific comments and our rewritten paragraphs under Overall Procedures (as well as earlier responses in this note).

- The authors do not explain why they chose a you-tube playlist as a control (page 4).

We employed a selected playlist that required similar scrolling actions used when viewing Facebook or Instagram, and short videos that were designed to not provoke any kind of response.

This is an appropriate control because it closely mirrors cell-phone use to social media viewing, which is the activity we wished to assess. We have added text to this effect in the manuscript.

- The methods section does not explain why the authors used salivary cortisol and heart rate as a sign of activation of the sympathetic system and HPA axis and as a physiological indicator of stress (briefly provided in Discussion, page 10).

- On page 6, The authors should clarify their choices regarding the physiological measures. They are essential info to support the interpretation of the data.

We have put these two comments together here because we believe they reflect the same concern. We have added information about these choices in the introduction and the abstract and have included relevant references. We are hopeful that this reviewer will find that these changes clarify the choices made. The references cited provide further detail into the physiology of the stress response, and we chose to limit detailed explanation to maintain focus on our study.

- The use of objective and self-report measures is a strength of this experiment. However, the manuscript does not mention which arm the heart rate monitor was placed on (and it could impact the accuracy of data collection) (page 3).

We understand that there may be differences in heart rate between an individual’s arm, but we did not move the monitor during the study period. We assume that arm response is consistent across social media and YouTube viewing (see response to why we chose YouTube as a control) so that any bias that is introduced by placing the monitor on (say) a dominant arm will be present for both treatment responses. We analyze the data in a repeated measures framework, and study differences in response, not absolute response. We have added detail about heart monitor placement in the article in response to specific reviewer comments.

- The study included a survey at the end of the experiment (page 4). Since there are divergent approaches and opinions on this, it could be helpful to justify this methodological choice (the only explanation is given at the end of the paper, in Appendix A, page 19).

We wanted to ensure that any stress induced by the instruments themselves would not be reflected in our treatments. We mention this reasoning now in the text in response to reviewer comments.

- As far as my knowledge goes, the statistical analysis appeared appropriate to the study objective. However, on page 7, the authors write, "Because of the period effect", as if the readers already know about this effect. It was only mentioned in the abstract.

Again, we are hopeful that the revised paragraphs about the design make this concern negligeable. We have added definitions specific to this study in our description of the study design and have further information in the table responding to specific concerns.

3. Limitations and future perspectives: Throughout the manuscript, the authors have listed a series of limitations (e.g., page 3 Participants, page 11 Discussion, page 19 Appendix A) that should be reported in this paragraph. Also, considering the fascinating findings and topic of the study, the list of "future research perspectives" (page 12) could be more detailed (e.g., page 9 Results; page 11 Discussion).

We have re-titled the Limitations section to “Limitations and Future Directions” to identify that some of these limitations do indeed represent future research opportunities. We have specified in this section that our inferences are limited to those who participated in our study, and thus we acknowledge that excluded populations (e.g. pregnant women and those with Cushings) are not considered here.

4. Citations and References:

- As the PLOS ONE webpage "Submission Guidelines" states, this should be titled "Reference list" (instead of "Literature cited") and citations should be listed in Vancouver style.

This has been corrected to read “References” and we have employed Vancouver style.

- The authors report several inferences on psychological outcomes (Page 11, self-esteem and social comparison related to feelings of challenge and threat; Facebook and Instagram perceived as benign; threatening posts undergoing cognitive process so they would feel harmless; page 12, social media use as coping mechanisms; activation of the parasympathetic system caused by "sitting still and scrolling through content"). However, the study did not include or measure these parameters. Because unsupported by the measurements or data collected, it would be safe to accompany those assumptions with adequate literature appropriately cited.

We agree with the reviewer that these statements needed to be identified as either supposition or opportunities for further research, and we have changed the wording slightly in the Limitations and Future Directions section to reflect this and to soften the statements.

5. Tables & Figures: Overall, figures and tables are easy to interpret and well structured. However, some details may be improved:

- Table 1 (page 2), some words are underlined. The authors should clarify why in the manuscript or the table description.

The studies were initially underlined because they suggest opposite findings, but we agree that out of context and without explanation these markings make no sense. They have been removed.

- The manuscript states that Figure 1 (page 4) reports the "abbreviations used throughout this article". The study design is extensively drawn, but the figure does not include abbreviations.

We changed “abbreviations” in the text to “identifying terms” (“Baseline” and “Final” are clearly not abbreviations) and explain those terms in the figure caption.

- Page 5 is blank, and a reason should be provided for this choice.

We failed to notice that the file conversion added a blank page. We have deleted this blank page from our manuscript.

- The authors describe Figure 2 in the manuscript as reporting "all four periods" (page 8), baseline-T1-interim-T2. However, Figure 2 (page 9) shows the four points in time only for the heart rate data, not the cortisol data. This difference should be clarified.

We apologize that we failed to catch this rather obvious difference. A statement has been added to the legend in Fig. 2 in order to clarify the point above. The sentence now reads “Raw cortisol values are plotted *and reflect titers at baseline and during the two 20-minute treatment periods (T1 and T2).”*

**Reviewer #2**: First, it is important to congratulate the authors for the original work submitted. The manuscript addresses an important field of research and a trending topic in the area. The research question and hypothesis made by the authors will fill a gap in the current literature. Authors took a multidisciplinary approach, considering not only physiological responses but also related psychosocial parameters using validated and well-established assessments. Having a more detailed picture of the phenomenon investigated. Statistical approach and analyses performed seemed appropriate and well executed.

However, there are several major and minor aspects that the authors should resolve. The title proposed for the current work is a generalization, it is strongly recommended to change it, specifying the length of the social media use and the physiological stress parameters investigated. It is a bit confusing how the experimental and control condition/treatments are well defined in the research question, but later described both experimental conditions. More clarity and consistency are needed in relation to the use of the following terms: experimental treatment/condition, experimental/treatment/condition order, treatment/condition effect, and period effect. It is not very clear the rationale for using 20-minute treatment periods interspersed by a 5-min interim period. The length of the treatment has not been considered as a limiting factor of the current study. The appropriateness of the chosen control treatment could be debated. An alternative study design, adding a control group (no cell phone) could have been proposed. When assessing the results and future implications of the current study, the length of the treatment used should be always specified in the text. Otherwise, the term "social media use" is quite general, and we don't know if your results can be extrapolated to other treatment durations.

We appreciate that these concerns have improved the manuscript overall and believe that all of these concerns were identified in comments by this reviewer, which have been addressed. Please see the table for Reviewer 2 for specific responses to these concerns.

Reviewer 1

| *Page* | *Com-ment Number* | *Comment* | *Response* |
| --- | --- | --- | --- |
| 7 | 1 | specify how (salvary cortisol levels) | We have added this clarification to the abstract. |
| 7 | 2 | before, during and after | We have added clarification to the abstract to indicate that heart rate was measured before & during cell phone treatments and that cortisol was measured before and after each cell phone treatment. |
| 7 | 3 | what do you mean by "controllede"? do you explain it further in the manuscript? | We have changed "controlled" to "pre-selected" hoping that provides clarity consistent with the rest of the manuscript. |
| 7 | 4 | Is period the right term? Wouldn't it be better time or duration? never mentioned in the manuscript | We appreciate that this word is new in the abstract, but as it is the abstract, we chose to leave it. We feel that the rest of the sentence clarifies the conclusion of the manuscript. We do discuss, in some detail, the period effect in the statistical analysis and the conclusions. |
| 7 | 5 | WordReference definition: to cause a feeling of anger in; annoy or exasperate; to arouse; to trigger. Different meaning from INCREASE or DETERMINE | We have replaced "provoke" with "elicit" to soften the language. |
| 7 | 6 | Afifi * They talk about "rise and increase of the physiological responses" | Thank you for your knowledge of the literature. We have used these references to support that social media may increase psycho-social stress, which is the focus of this paragraph. Therefore, we do not believe we need to discuss all findings from these articles at this point in our manuscript. |
| 7 | 7 | They write "augmenting" |  |
| 7 | 8 | They specify "delay or impair" stress recovery |  |
| 7 | 9 | This verb has a positive connotation. WordReference: to help or encourage to exist or flourish. Maybe better looking for synonyms | Thank you, we have used the word "elicits" in place of "promotes", which may fit better in the context of the sentence. |
| 7 | 10 | This noun has a negative connotation, usually not associated with a verb like "promote" |  |
| 7 | 11 | WordReference: From the verb curate.  1. a member of the clergy employed to assist a rector or vicar. | We understand that this word has multiple meanings, but in this case, we view curated to mean carefully chosen (https://www.merriam-webster.com/dictionary/curated) and have chosen to leave the wording as-is. |
| 8 | 1 | Study Rationale | We note that the reviewer had some thought about these highlighted sections, but we did not see the comments and are unsure how to respond. We have left the highlighted sentences intact. |
| 8 | 2 | Hypothesis? |  |
| 8 | 3 | Why underlined? | We have removed the underlining to avoid explaining the differences between these two studies - in one, testing followed a stress instrument, and in the other, the stress instrument followed testing. Interested readers will note this different without the underlining. |
| 8 | 4 | Why underlined? |  |
| 8 | 5 | Although the protocol asks participants to use social media as "typical use", it is a single bout measure. The world "routine" may indicate "repeated in a long time" | We have changed "routine" to "typical" in accordance with the title of the paper. Thank you for suggesting this consistency. |
| 8 | 6 | These details should be cleared in the abstract * There should be a sentence and citation that connects sympathetic nervous system and HPA axis to stress: the manuscript does not state that why they measured the physiological response as aactivation of those regions (and non-neuroscientists, like IT experts working on social media platforms, may not have this knowledge). | We appreciate the suggestion to clarify physiological indicators of stress, and we have added this wording to the abstract. |
| 9 | 4 | It is not clear the intervention process here: first, it says 1 group per day (15 on the 1st, 21 on the 2nd, 23 on the 3rd); them, it says "on a given day, all participated simultaneaously" | We understand the confusion created and hope that our small change improves understanding. The sentence has been updated to read "On each of the three days, all participated simultaneously and were randomly assigned to each experimental group order." |
| 9 | 5 | While the authors specify the "missing data for physiological measurements, there is no reference to the missing data for the psychological tools. Reading the manuscript, you can find these info separatedly, in the Appendix A. | We recognize what the reviewer sees as an inconsistency here. There were very few missing data in the surveys, and we imputed these values (as described in the appendix) to allow calculation of the scales. As a result, all participants had complete survey scale data, and so there were no missing data for these values. |
| 9 | 6 | Relative only to "non-evocative content on youtube"? What about the other experimental condition (social media)? | We appreciate that explanation of the experimental design leads to confusion and thank the reviewer for their patience. We have added definitions to the control and experimental conditions and added more detail on the crossover study design as well. This is now two paragraphs, and we are hopeful that the reviewer will find this a more clear description of the study design. |
| 9 | 7 | It does not specify which arm. It seems the best arm is the non-dominant one, and this might make a difference on the accuracy of data collection. | We did not choose a dominant or non-dominant arm for the heart rate monitor. Each participants wore the heart rate monitors on the same arm for the duration of our study. We assume that if there are differences in heart rate measures by arm, they will be consistent throughout the study duration. As we use repeated measures to analyze the data, we do not expect our results to be influenced by this. |
| 10 | 1 | So it is not a routine use because it was measured only once | The author is correct, and we have fixed this issue throughout the manuscript, eliminating the word "routine" and selecting different, more appropriate words. |
| 10 | 2 | The author do not explain why they chose a you-tube playlist as a control (is the literature described any calming effects related to this tool that should somehow counteract the expected stressing effects of social media use?) | We thank the reviewer for this observation; we re-wrote our explanation in this paragraph to reflect the similarities in activities between our YouTube list (participants had to scroll through the list to select the videos, which were all quite short) and Facebook / Instagram (participants scroll through content, pausing to view videos and read posts). |
| 10 | 3 | If the "use" duration did not change, how do the authors state the "period had an impact? | We are hopeful that our change to the first paragraph in the "Overall Procedures" section has clarified that the period refers to either the first 20-minute cell-phone activity (Social Media or YouTube) or the second 20-minute cell-phone activity (Social Media or YouTube). |
| 10 | 4 | The authors speciffy they administred the survey at the end of the experiment, but did not say why. Since there are divergent approaches and opinions on this, it could be healpful to justify this methodological choice. | Thank you for this suggestion. We have added an explanatory sentence in this paragraph. We were concerned that the surveys themselves may influence the stress level of the participants, so we put this collection at the end of the data collection period. |
| 10 | 5 | It seems that FIgure 1 does not list any abbreviations. | We appreciate the detail. We have changed "abbreviations" to "identifying terms" in the text (in addition to replacing "Experimental" with "20-minute" in the figure itself. |
| 11 | 1 | Is there a reason for this blank page here? | Thank you, we failed to notice the formatting change on conversion from a word document to a pdf document. |
| 12 | 4 | This sentence is not clear and makes it hard to understand what the author intended to do. | We have removed the detail about additional calculations and the paragraph now reads more clearly. In response to another reviewer, we have retained some of this information, but we hope this simplifies the text. |
| 12 | 5 | There should be a reference here. | Thank you for this suggestion. We have modified the sentence for clarity and added references. |
| 13 | 1 | The author mention a "strong periodo effect" here, for the first time. It is not clear what it means and if it is a result of the experiment. | Thank you for noticing that the results are in the methods section. We have re-written the paragraph in the statistical analysis section. The statistically significant period effect informed the analytical process. We hope that the reviewer finds this wording clarifies the concern. |
| 14 | 1 | Which group? Is this reported in discussion/conclusions? | Thank you for noting this lack of detail. The statement now reads, "We noted that Facebook Intensity was slightly higher in the Social Media than YouTube group.". |
| 14 | 2 | noted* | We have incorporated this change into the re-written statement. Thank you. |
| 14 | 3 | It seems like there was a decrese in the physiological response, and this decrease was related to the duration of the experiment (physiological response decreased throughout the intervention). Is this right? Also, this effect was significant in both groups, which means the effect was probably due to the time of using either of the experimental conditions, not to a specific condition. Is this correct? | The reviewer very clearly states the conclusions of our study. We truly hope that the clarification of the study design makes this easier to see, rather than having readers surmise this on their own! Yes, both statements are correct, and represent our key results. |
| 14 | 6 | This should be said before the line about "The preiod effect..." above. | We agree that the order of statements certainly buries the lead. However, in a cross-over study, before one can conclude a treatment effect, one must examine carryover, order, and period effects. The presence of any one of these alters how conclusions are made from the model. Therefore we (sadly) feel it is most appropriate to leave the order of the statements intact. |
| 14 | 7 | between what? | We agree that this was not clear without examining the Analysis of Variance table, and we have added "between order and period" to the sentence. In addition, we updated the sentence in the methods about how we use the model to estimate the treatment effect from the order term. |
| 15 | 1 | Figure 2 shows a difference between T0 and T1 both in heart rate and cortisol level for the group "Social Media then Youtube". Do the author mean this difference between T0 and T1 scores is not significant? So there is no actual "period effect"? * Why Figure 2 does not show the interm time point for the cortisol levels? | In answering these questions, we assume the reviewer means T1 and T2, and not T0 and T1. The point estimates for the "Social Media then YouTube" group do decline from T1 to T2 for both mean heart rate and cortisol, and from Baseline to T2 for both measures as well. (Other temporal differences for heart rate are also present). However, these differences exist for both groups. Our model indicates a period effect (p < 0.001 for both), meaning that successive measures are generally lower for both heart rate and cortisol as the study progressed, whether the participants were viewing YouTube or their Social Media.  Cortisol collected during the Interim and Final periods reflect the cortisol values from the 20-minute periods preceding them, not the exact period the measures were taken. We have edited the legend in Fig 1 to highlight this point. |
| 15 | 2 | Nontheless, it may be a further research perspective. | We agree with the reviewer that there might be more to unpack here, but this difference was present only at Baseline and was not reflected at the other time points, so we did not choose to pursue it further. |
| 16 | 1 | This explanation might be reported earlier in the manuscript to explain why physiological stress is measure as sympathetic nervous system and HPA axis activation (although, it does not mention the role of "cortisol"). | Thank you for this attention to detail. We believe it helps explain our results, and therefore belongs in the discussion. We have added some detail in the abstract and the introduction to connect physiological stress to our specific measures. |
| 17 | 2 | Is this inference supported by any literature? How are self-esteem and social comparison related to feelings of challange and threat? The authors need to justify their assumptions with evidence in here. | The reviewer is correct that we lack evidence for that statement; we have added "we surmise" before the statement to make this clear. |
| 17 | 3 | If this is the supporting evidence for the inferences provided above, it should be described beforehand. | We understand why the reviewer may see this, but it is not evidence for the inferences provided above. Therefore, we have made no clarifying statements in response. |
| 17 | 4 | Again, the study did not include any feelings/emotional scale so, unless some literature is provide, these inferences seem far from the collected data. | We agree with the reviewer and have softened these statements from both of these comments to indicate that they are beyond the scope of the collected and analyzed data. |
| 17 | 5 | These are both future research pespectives; not assumptions made on the data collected and analysed here. |  |
| 18 | 1 | Throughout the manuscript, the authors have listed a series of limitations (highlighted in purple here) that have not been reported in this paragraph. | We appreciate that the reviewer has identified limitations that may not be immediately recognizably identified in the limitations section. Regarding the exclusion of pregnant women and those with Cushing's syndrome, these conditions are known to inflate cortisol, and in response to reviewer comments we have added references for this. This quasi-experimental study was limited to those who agreed to participate, resulting in the gender and age balance described. Our first sentence of the limitations generally addresses the inability to make inferences beyond those who are represented by our participants. The final highlighted statement, referring to participating in research eliciting its own stress, is addressed within that paragraph, and we do not therefore recognize this as a limitation. |
| 18 | 2 | There is no actual reference to any "future research perspectives". This sentence gets closer to a suggestion for further developments. However, there are at least two more above in the manuscript. | We agree with the reviewer and have renamed the Limitations section to Limitations and future directions. |
| 18 | 3 | "Unconstrained" how? In time or content or type or platform? | We agree with the reviewer and have changed "unconstrained social media" to "physiological and psychological effects of social media" and hope this clarifies the intent of our sentence. |
| 18 | 4 | Again, here coping is mentioned. However, it is one more psychological construct that has not been mentioned, measured, cited or treated in any way during the manuscript. (no evidence, no literature that connects the actual results of the study to the coping mechanisms) | We agree with the reviewer and have changed "as a stress coping tool" to "mitigating the effects of stress" and hope this clarifies the intent of our sentence. |
| 18 | 5 | How do the author state that "sitting still and scrolling through content" may cause the activation of the parasympathetic system? They have not measured or cited any outcome related to it in the rest of the manuscript. | The reviewer points out that this is the first use of the parasympathetic nervous system, and we have re-framed this last sentence in terms of the sympathetic nervous system to make this point clearer. |
| 19 | 1 | As the PLOS ONE webpage "Submission Guidelines" states, this should be titled "Reference list" and references should be listed in Vancouver style, which means each citation should have a number assigned in order that they appear in the manuscript. | We have made this correction, thank you. |
| 25 | 1 | This and the following one are important "limitations" that should be listed accordingly. | We thank the reviewer for this careful consideration. In the cases identified in these two comments, we imputed the data for these missing survey items. This allowed scale summaries to be calculated for these participants and for all of our surveys, we had complete responses. We do not understand how this represents a limitation to our inferences and conclusions and thus we have not addressed it as such. |
| 25 | 3 | This should be stated in methods section when describing the tools and the approach (as said in the relative comment above). | In response to this concern, we have added two statements about this in the methods. We have added a statement about why the surveys were given after the treatments, and in the Psychological Measures sub-section, we stated that the Perceived Stress Scale was given last to avoid its influencing other survey responses. |

omment #

Comment #

Reviewer 2

| *Page* | *Com-ment Number* | *Comment* | *Response* |
| --- | --- | --- | --- |
| 7 | 1 | Since your study assessed the use of social media for 20 min only, and the physiological stress response parameters investigated were heart rate and salivary cortisol, I would strongly recommend to rephrase the title making sure that these aspects (20 min use, heart rate, and salivary cortisol) are included. | We have changed the title to reflect these concerns. We have used "short term" rather than 20-minutes for clarity. We have also changed "provokes" to "elicits" in response to a different reviewer. |
| 7 | 3 | Please add the period of time referred to the smartphone use. (i.e., 40 min). Longer durations have not been investigated in this study. Probably it is also worthy to mention here that the use of social media and/or YouTube. Other activities that can be done with an smartphone (playing video games, listening to music) were not investigated in this study. | Thank you for this suggestion. We have added this clarifying wording to the abstract. |
| 7 | 5 | Since the population tested were between 13 and 55 years old. A reference for adults is needed here. | We have added more information and included references. |
| 8 | 2 | From Table 1 it seems that cortisol findings are in the same direction: the more use of social media the higher the greater the cortisol response. Only 2 studies reported heart rate response, and arterial/blood pressure was not measured in your study. Therefore, I would separate the findings (cortisol, heart rate) | We appreciate attention to this detail. We do not agree that the cortisol findings are in the same direction, nor are the heart-related measures. We feel that a table delineating these inconsistent findings would be clearer than extensive text. We have changed the word "equivocal" to "not consistent" to clarify. |
| 9 | 1 | To provide the reader with some context, please briefly explain the reasons for excluding this specific populations. | Thank you for this suggestion. To clarify these exclusions, we have rewritten the sentence (now two sentences) and added references. |
| 9 | 3 | Were participants screened for any potential factor that could affect heart rate at rest? (i.e., caffeine, sleep, medication) | The reviewer makes an important point that some substances raise heart rate. However we believe whether artificially elevated or not, the effect would last the duration of study , without altering the response to social media or YouTube viewing than would otherwise have occurred. The repeated measures analysis will account for this in the analyses, assuming that our assumption about effect throughout study duration is correct. |
| 9 | 6 | If known and possible, please report the cross-reactivity value here, so the reader can compare later with your results (19.2% for dexamethasone; less than 0.6% for others). | We thank the reviewer for pointing this out. We have added the cross-reactivity to the text (less than 0.015%). |
| 9 | 7 | Please add some context here about the importance to standardize the time of the day for testing due to the cortisol circadian rhythm. | We have added the clarification and added a reference also. |
| 9 | 9 | First author surname is "Milliken" not "Millikin". | Thank you, this misspelling has been corrected. |
| 9 | 12 | Your experimental hypothesis is "that social media use would impart some degree of psychosocial stress that would activate the physiological stress response more so than our control treatment (non-evocative You-tube playlist)".  Therefore, you have one experimental condition (social media use) vs. a control condition (YouTube), not two experimental conditions, if I understood well. It is confusing, from a reader perspective, due to the fact that the study is based on two groups, depending on the treatment order.  Please rephrase the highlighted section accordingly. | We address these concerns together, and appreciate the effort made by the reviewer to parse the complexities of the cross-over design. We have changed the wording to reflect "treatment" (as suggested by a later comment) and use this to collectively refer to an experimental treatment (viewing social media) and a control treatment (viewing a YouTube playlist). We re-wrote the entire paragraph to clarify treatment vs. treatment order, and period vs. treatment and treatment order. In addition to concern about misinterpreting treatment order, as the reviewer notes, we are also concerned about misinterpreting a period effect as a treatment effect. These ideas are now in two paragraphs, and we are hopeful this clarifies the experimental process. |
| 9 | 14 | If I understood well, the point here is to mention that the randomization of conditions order ensured that an order effect would not be interpreted as a treatment effect.   Please rephrase the highlighted section accordingly. Because it is a bit misleading, you have one experimental condition (social media) and one control condition (YouTube). |  |
| 9 | 16 | Were the participants given any instructions about how to place the sensor in their arms properly?  According to the manufacturer, the sensor must be in firm skin contact, but not too tight to prevent blood flow. To get accurate readings placing the sensor in a tattooed area should be avoided.  Source: https://support.polar.com/e_manuals/OH1/Polar_OH1_user_manual_English/manual.pdf | We realize this was not mentioned and thank the reviewer for catching this. We have added the phrase "by the research team" to indicate that participants were not the ones placing the heart rate monitor and edited the following sentence for clarity. |
| 10 | 1 | YouTube treatment = control condition | We appreciate the reviewer's concerns about accurate reflection of experiment and control conditions. In line with this reviewer's comment 9, and comments 12 & 14 on the previous page, we have opted to use "treatment" rather than "condition" throughout and have otherwise retained the original language from the article. |
| 10 | 3 | Social media treatment = experimental condition. |  |
| 10 | 5 | It could be expected that scrolling behaviour would be different between Facebook/Instagram and YouTube due to the longer duration of the stimulus (i.e., videos) in the latter one. Please further explain why, in the authors' opinion, is similar. | We thank the reviewer for this observation; we re-wrote our explanation in this paragraph to reflect the similarities in activities between our YouTube list (participants had to scroll through the list to select the videos, which were all quite short) and Facebook / Instagram (participants scroll through content, pausing to view videos and read posts). |
| 10 | 7 | Please further explain the reason to choose a 5-minute interim period. Is there any other research or evidence suggesting this? Would it be possible that 5 minutes is not long enough to detect changes in salivary cortisol levels caused by the intervention? | We thank the reviewer for identifying this lack of clarity on the methods. We have added the reason for the timing in an additional sentence in the paragraph. Evidence suggests that cortisol can peak 20 minutes after the treatment, so it would be reflected in the cortisol sample collected during the interim period. Regarding the five-minute time for collection, it is simply what we used to standardize data collection and to ensure all cortisol samples could be collected before starting all participants on the second 20-minute period. |
| 10 | 9 | In order to improve readability, it may be worthy to use consistently the term "condition". Or "treatment" instead if you prefer. Using both alternatively may confuse the reader. | Thank you for this suggestion, which has added a great deal of clarity to the writing throughout. We have left the original language, referring to treatments, in the manuscript. |
| 10 | 10 | I would say "completed both treatments but in a random order" instead of "viewed". | We have made this wording change in the manuscript, thank you for the suggestion. |
| 10 | 13 | Have you considered whether 20-min experimental periods of smartphone use may not be long enough to induce any stress? Is there any piece of research in the literature about this? For example, in mental fatigue research, there is evidence suggesting that the length of the trial may induce (or not) mental fatigue. | The reviewer is correct that 20 minutes could be insufficient to generate stress, and we have added a sentence in the limitations to address this possibility. We chose the 20-minute length because it gave sufficient time to generate a cortisol response to perceived stress but was the longest that we could reasonably ask people to participate. |
| 12 | 1 | I was wondering whether the analysis of the heart rate response during the baseline period was an average of the last 5 minutes.   As written previously in "overall procedures" section participants were sat quietly for 5-15 minutes (depending on order of entry). An average of the short end of the range (5 min) seems more appropriate | Thank you for catching this.  We have removed the first sentence of this paragraph, and added the text, "We calculated the average heart rate from the final five minutes prior to T1 and T2 for the Baseline and Interim periods, respectively." |
| 12 | 3 | I would remove then the previous sentence above about median. 95th percentile, first and last five minutes of the activity.  However, it may be worthy to compare the last 5 minutes of the baseline vs. the last five minutes of the experimental and control | We have removed the statement about the other calculations, however, that includes the comparison of the last five minutes of the baseline vs. the last five minutes of the experimental periods. The entire paragraph now reads: “Using time records, heart rate data were encoded to match the study periods described in Fig 1. We calculated the average heart rate from the final five minutes prior to T1 and T2, and identified them as the Baseline and Interim periods, respectively. For T1 and T2, we used the average heart rate from the full 20 minutes of each period. Unreported comparisons based on the last five minutes of all periods yielded no change in the results.” |
| 12 | 5 | Is there any reference supporting the use of this to stabilize variance? | Thank you for reminding us about this missing reference. We have inserted a reference (Kutner et. al, 2005) and added it to the References. |
| 13 | 1 | First author surname is "Milliken" not "Millikin". | Thank you, this misspelling has been corrected. |
| 13 | 3 | "cell phone" for consistency. Also to simplify, it could be suggested to use the treatment order instead. | Thank you for noting these inconsistencies. The phrase has been changed to read "We then added each potential moderator (sex, age, and the four psychological measures) to the model to examine whether there was a change in the observed relationship between the treatments and physiological stress response;". |
| 13 | 5 | Any reference for the use of 25 years old as a "threshold" age to define "digital natives"? | We understand the author's concern, and we do not see a threshold in the literature. Here is the rationale we used: Facebook began in 2004, and the first smartphones became available in 2007. If a 13-year old got their first smart phone in 2007, they would be 25 years old when our study was conducted in 2019. This seemed like a reasonable cut off to us. |
| 14 | 1 | above by the authors: "Statistical significance was defined as p (or adjusted p) ≤ 0.05."  Therefore, it is not statistically significant, irrespectively it could be considered "marginally" or not. | We recognize this inconsistency. The statement now reads "We noted that Facebook Intensity was slightly higher in the Social Media than YouTube group." We have also removed the statement defining statistical significance with a hard cut-off. |
| 14 | 3 | In order to improve readability, it may be suggested to report N values in each of the variable name columns. | This change improves the table; thank you for the suggestion. |
| 15 | 1 | Please report in the axis title the name of the variable plotted and the units of measure, instead of doing that in the graph title.  Additionally, please add in the Figure 2 text that the data plotted are mean +/- Standard Error.  Furthermore, could you please explain the reason for choosing Standard Error instead of Standard Deviation? | Thank you for these clarifying suggestions. The axes labels are now on the Y axes as suggested, and we have added the requested text in the caption. Both the standard error and standard deviation reflect the degree of precision in the mean value; standard deviation is large enough that it can have a distorting effect on the plot’s y axes. Thus, we selected standard error. |
| 16 | 1 | 20 minutes of social media use | We have added 20 minutes to the sentence, thank you for this suggestion. |
| 16 | 3 | Would it be possible that the intervention (20-40 min) was not long enough to have detrimental effects?   Would it be possible that a different control treatment (resting without a cellphone) would have shown different results?  Would it be possible that the salivary cortisol response may be delayed, so your salivary cortisol measure was unable to detect changes that may have occured later after the intervention? | We recognize the points that the reviewer is asking us to address. We have statements in the limitations section about the effects of the delay, and about the length of the exposure.  We think there are many possible ways to control for Social Media use in this study, and the choice of controls depends upon the question being asked. In this case, we believe a different control, such as resting without a cell-phone, would offer relevance if we had known in advance what our results were. That would have required recruiting additional participants for a third treatment and added to the length of the study. We stand behind our choice of control to separate out how different cell phone uses are associated with a physiological stress response. |
| 17 | 1 | Please add the duration used in your study. | We have added the duration to the statement. |
| 17 | 3 | Probably here is interesting to mention the lack of a control treatment with no cell phone. | We appreciate that the reviewer may believe the study is under-controlled because a non-smartphone, rest-only treatment was not given. We reiterate that we do not believe such a treatment is a sufficient control to study the association between social media use and physiological stress response. We further clarified why the YouTube treatment was a suitable control in our methods section. |
| 18 | 1 | Please, approach other limiting factors here, such as the duration of the intervention, a potential delay in cortisol response, and the control treatment chosen. | We have added a final sentence to the first paragraph in the limitations section addressing duration of the intervention and the potential delay in cortisol response. We have addressed the concern about control treatments elsewhere. |
| 18 | 3 | Please add the period of time used in the study. | We agree with the reviewer and have added the 20 minute time reference. |
| 19 | 1 | Please use "References" instead. | We have made this correction, thank you. |
