# Supplementary material for: Social media does not elicit a physiological stress response as measured by heart rate and salivary cortisol over 20-minute sessions of cell phone use
Source: PLoS One. 2024 Apr 3;19(4):e0298553. doi: 10.1371/journal.pone.0298553 (PMC10990243; doi:10.1371/journal.pone.0298553)
Supplement: Attachment — Submitted filename: comments & responses_31Oct_SO.docx [file pone.0298553.s007.docx]

*Editor’s and Reviewer’s comments are in black text, and authors’ responses to editor’s and reviewer’s comments are in red. Please note that page references may be slightly off, depending on which “track changes” format is being used by the reader; they should be close, regardless.*

*Also, please note that we added a “B” to our existing text that directs readers to an Appendix. With the addition of supplementary material, we now have an Appendix A and an Appendix B.*

*EDITOR’S COMMENTS:*

(1) Significantly improve the wording of several paragraphs of the brief.

We believe this refers to the comments from Reviewer #2 and have addressed them below.

(2) I suggest incorporating an explanatory figure, such as a flow chart, regarding the inclusion of participants.

We have provided such a figure below, and if it were to be included, it should appear as Figure 1 following the second paragraph under “Participants & overall procedures,” with the final sentence reading “…completing all questionnaires (see Figure 1).” This keeps it with text already describing our participants. However, this placement would precede the current Figure 1, the design schematic, and may increase confusion, rather than reduce it.

If you feel that the numbers of participants are opaque without this figure, then we would prefer to clarify this in the writing. The figure above mimics a participant flow diagram from the STROBE checklist, and we do not otherwise claim to be in STROBE compliance. (We are close.) The inclusion of this figure may invite criticism from readership beyond our reviewers that expect a STROBE statement in the presence of this figure.

(3) It is strongly requested to include information regarding the cortisol assay used, as well as the conditions under which the determinations were made.

We have added additional information regarding the cortisol assay in the *Material & methods* section (under *Physiological measures*) on pgs.10 & 11.

(4) In accordance with the above, it is requested to provide more detailed information regarding the interpretation of the cortisol assay results.

Additional information has been added to the methods sections (see above). It was unclear if additional information was requested regarding our interpretation of the cortisol data in the results section. As a reference was made to the previous comment, we assumed this comment was directed at more detail in the methods section (which we have added).

(5) For a better understanding, I suggest, as far as possible, to incorporate as supplementary material, a list with links to youtube videos used.

Text was added at to pg. 9 (1^st^ paragraph after Fig. 1) to direct readers to the Appendix for a list of and links to videos in this playlist. In addition, a list and links were added to our supplementary materials as Appendix A (pgs. 31 & 32).

*REVIEWER #2 COMMENTS:*

**Page 7. Comment number 7:** For the sake of clarity, the authors propose the use of “short-term” instead of the intervention time (20 min) used in the study. The new title proposed is not acceptable for this reviewer, unless further evidence is provided showing that the typical use of social media in the population studied is close to their intervention time. Otherwise, it is strongly recommended by this reviewer to find a more accurate title, for the sake of good practice.

We hope that our new title, *Social media does not elicit a physiological stress response as measured by heart rate and cortisol over 20-minute sessions of cell phone use*, is both accurate and will engage potential readers.

**Page 8. Comment number 2:** Only 1 out of the 6 studies presented in the table did not report a difference in cortisol and social media usage. The following 4 investigations have shown results in the same direction: cortisol levels and social media usage are positively associated. (Morin-Major et al. (2016): the higher the diurnal cortisol level the higher the number of Facebook friends; Rus & Tiemensma (2017): the higher the cortisol levels, the higher the social media use; Afifi et al. (2018): the greater the cortisol awakening response, the more frequent use of social media; and Shafi et al. (2021): the higher the cortisol levels, the more usage of social media). For this reviewer, 1 study out of 6 is not enough evidence to claim that findings are inconsistent, when 4 out of 6 have shown the same direction of response. Please, either provide more references showing no differences and/or negative associations between cortisol levels and social media usage or avoid using inconsistent results as an argument here.

Thank you for noticing the possibility of misinterpreting the statement made about physiological variables. While the majority of referenced studies do indicate that cortisol increases with social media use, other physiological variables that serve as indicators of physiological stress are less consistent in this context. Of the physiological variables in the referenced studies, 5 are positively associated, 2 are negatively associated, and 2 show no relationship with social media usage. In order to highlight that we are discussing several physiological variables, we have added text on pg. 3 (end of 3^rd^ paragraph of *Introduction*). We hope that this clarifies the intent of the statement.

**Page 9. Comment number 3:** Assumptions are made as part of the introduction. Lack of control of potential factors that could influence one of the variables investigated should be mentioned in the section about potential limitations of the study. This reviewer disagrees with the assumption made by the authors that the effects will last for the entire duration of the study. As an example of that: participant A took a coffee at 6 AM; participant B took a coffee at 11:30 AM. This issue is not being solved by using a repeated measures design, as mentioned by the authors in their response. By the time the participants were tested (12:30 PM) the effect of caffeine on their experiment is clearly different from participant A (very likely no effect) to participant B (very likely to have an ergogenic effect). We could hypothesize that participant A and B took their coffees at the same time of the day every day, so if they were both involved in two separate testing sessions, we could agree that caffeine is very likely to have the same effect during both experimental days. However, the investigation presented by the authors uses a single-day design. Hence why this reviewer is making a big emphasis on the importance of potential factors that may have an influence or their results. It is understandable that the authors may have not recognized this point when designing the study. But it is expected, at least, a mention in the potential limitations of their experiment.

We have added text to our *Limitation & future directions* section (end of 1^st^ paragraph) to acknowledge that events that occurred prior to our study could have influenced participants’ responses to our cell phone treatments. We hope that this addition appropriately addresses the reviewer’s concern.

**Page 9. Comment number 7:** if possible, add standard deviation and the range of the start of the testing. It is good practice to give the reader an idea of the accuracy of the starting time, that usually might be delayed due to logistical issues. Also, it would give additional information regarding potential outliers due to a very late start, compared to the average. However, this reviewer understands that this could not be taken into consideration when collecting the data.

Thank you for pointing out that our methods were not clear regarding our sampling protocol. While we had mentioned that samples were collected simultaneously in the 2^nd^ paragraph of *Participants and overall procedures*, this is easy to miss. We have added additional text on pg.7, (immediately prior to Fig. 1) restating that samples were collected simultaneously on a given day and to note that among the 3 days of testing, sampling times varied by up to 8 minutes.

**Page 14. Comment number 1:** It is good practice to describe in the corresponding section about statistics the p value set up for significance. In most studies, defined as a value smaller than 0.05. Removing this from your manuscript is not acceptable for this reviewer.

As requested, we have defined statistical significance as p≤0.05 in the last paragraph of our *Material & methods* section. We also invite the reviewer to explore discussions surrounding best practices in the use of p-values in null hypothesis significance testing. Please see: <https://www.ncbi.nlm.nih.gov/pmc/articles/PMC5187603/> <https://magazine.amstat.org/blog/2021/08/01/task-force-statement-p-value/> <https://journals.sagepub.com/doi/10.1177/0956797611417632>
