# Supplementary material for: Social media does not elicit a physiological stress response as measured by heart rate and salivary cortisol over 20-minute sessions of cell phone use
Source: PLoS One. 2024 Apr 3;19(4):e0298553. doi: 10.1371/journal.pone.0298553 (PMC10990243; doi:10.1371/journal.pone.0298553)
Supplement: Attachment — Submitted filename: Response to Reviewers.docx [file pone.0298553.s008.docx]

Responses to Academic Editor

| Comment | Response |
| --- | --- |
|  |  |
| Abstract The article abstract provides an adequate overview of the study, which investigates the physiological response to stress, as measured by heart rate and salivary cortisol, in 20-minute sessions of mobile phone use, specifically in the context of social media use and a pre-selected YouTube playlist. While the abstract clearly sets out the scope and key findings of the study, it could benefit from a clearer articulation of its relevance in the broader context of current research on stress and social network use. | We have changed the first three sentences to reflect the broader context of current research: "The pervasive use of social media has raised concerns about its potential detrimental effects on physical and mental health. Others have demonstrated a relationship between social media use and anxiety, depression, and psychosocial stress. In light of these studies, we examined physiological indicators of stress (heart rate to measure autonomic nervous system activation and cortisol to assess activity of the hypothalamic-pituitary-adrenal axis) associated with social media use and investigated possible moderating influences of sex, age, and psychological parameters. " |
|  |  |
| State of the Art The state of the art discussed in the manuscript addresses the existing literature, but could be further elaborated to establish a stronger argument for the need for this research. Given the volume of previous work in the area of social networks and stress, a more detailed comparison with previous studies and a clear justification of how this study contributes to filling a gap in the literature would be beneficial (in my view it would simplify the reading). | We have addressed this gap in three places: We have re-written the end of the second paragraph in the introduction, to now read, "Taken together, the body of literature suggests that multiple factors related to social media contribute to its association with psychosocial stress. However, it is unclear whether social media use leads to stress or if stress leads to social media use. This study attempts to fill this gap in the literature by addressing whether short bouts of social media use evoke stress. " We have added a sentence to the end of the third paragraph: "This points to a more nuanced relationship between social media use and physiological and psychological stress; we feel that there is a need for further research in this area to help tease apart this relationship." Lastly, we point out that most studies focus on correlations in the last paragraph of the introduction, saying "Unlike these studies (Table 1) that examine correlations or social media’s influence on an acute social stressor (i.e. TSST), the focus of our controlled study is to evaluate the physiological response to a short-term bout of social media use . . ." We are hopeful that these additions will address the Academic Editor's concerns |
|  |  |
| Problematisation and Research Question The problematisation of the study, which investigates whether the use of social networks induces a physiological stress response, is relevant. However, the specific research question could be more clearly formulated to accurately reflect the focus of the study. | We have added text at the beginning of the last paragraph in the introduction, to re-state our hypotheses in a fashion more specific to the study. The paragraph, with the key additions underlined, now reads: "Unlike these studies (Table 1) that examine correlations or social media’s influence on an acute social stressor (i.e. TSST), the focus of our controlled study is to evaluate the physiological response to a short-term bout of social media use and indirectly takes into account the potential moderating effects of age, sex, and psychosocial factors." |
|  |  |
| Hypotheses and Objectives The hypothesis that social network use does not elicit a physiological stress response is adequately tested in the study. The aims of the study are clear, although they could benefit from greater specificity in terms of how the results could inform current understanding of the interaction between social network use and stress. | We have added additional sentences to the end of the last paragraph of the introduction to identify how the results could inform the relationship between social media use and stress: "We examine the physiological effects of short bouts of social media use because this is often how it is accessed (e.g., as we wait for a friend or take a break from another task). If this type of usage results in a stress response, then this could be one potential mechanism by which social media poses a health risk." |
|  |  |
| Methodology The methodology employs a crossover study design with an interesting approach to compare the treatment effect of mobile phone use on the physiological response to stress. However, there are significant limitations: | Responses to specific points are given below: |
| Sample Selection and Sample Size: The sample of 59 subjects spans a wide range of ages, which is positive, but how differences in age and sex might affect the results could be explored further. | Early readers of our manuscript suggested limiting the non-significant results, but we did analysis whether our results would be changed with either age or sex added to the model. We have incorporated these results into the final paragraph of the results section. The text now reads, "We separately added sex and age to our models examining the effects of order and period (as indicated by a 3-way interaction) and found that neither heart rate nor cortisol responses were moderated by participants’ sex (heart rate p = 0.361, cortisol p = 0.442) or age (heart rate = 0.545, cortisol p = 0.837). " |
|  |  |
| Controlling Variables: The study mentions the lack of control over the content of the social media viewed, which could introduce significant variability in the stress response. In addition, caffeine consumption and other dietary or environmental factors that could influence cortisol and heart rate measures are not adequately controlled for. | We have added caffeine and other factors more explicitly in our limitations section. This concern was brought up by a reviewer and so we felt it best to tackle this head-on. The second paragraph of the limitations section now reads, " Beyond excluding those who ate within one hour of our study from cortisol analyses, we did not control for food or beverage consumption or emotional state (e.g., caffeine consumption, hard exercise, exogenous stressors, or calming activities), prior to collecting our data. It is possible that such events could have altered participants’ responses to cell phone treatments throughout the duration of our measurements, and our analyses could not have controlled for this variation in response. However, even if such uncontrolled variables had an effect, it would not change our conclusion. If social media did provoke a stress response, then we would have seen either no change in or an increase in heart rate and cortisol across the duration of our study period and not the observed decrease in these biomarkers." |
|  |  |
| Discussion The discussion provides a detailed analysis of the findings, comparing them with previous studies and related theories. However, the discussion could be strengthened by further addressing how these findings contribute to the existing literature and what implications they have for understanding social media-related stress. | To tie our results back to the literature and identify implications for understanding social media-related stress, we have added three sentences to the discussion: The first paragraph of the discussion ends with, "Instead, our findings seem to lend credence to the suggestion (36,37) that accessing social media (and in the case of our study, YouTube) acts to assuage stress." We have slightly altered the first sentence in the second paragraph to read, "Nevertheless, despite the absence of a physiological stress response to our social media treatment, our findings do not preclude the possibility that social media use induces psychological stress as has been frequently posited", and added three additional citations. Finally we have added a concluding paragraph to the discussion, "Because cell phones and social media have only recently come into widespread use, much is left to learn about the social, psychological, and physiological implications of this novel technology. Previous studies have examined social media use with various methodologies (e.g., 13,20,39) and our study is unique in that it evaluated the physiological consequence of social media use using a robust cross-over design. Our findings add to a growing body of knowledge and imply that short-term bouts of cell phone use may not stimulate an acute physiological stress response." We are hopeful the Academic Editor agrees that these additions unify our findings with the current literature. |
|  |  |
| Conclusions The conclusions are consistent with the results, highlighting the lack of a significant physiological stress response associated with social media use. However, it would be beneficial if the conclusions also addressed the limitations of the study and suggestions for future research (although these have already been discussed). | We have rephrased our conclusions to be specific to our study participants, now saying "Recognizing the limitations of this study, we found that 20 minutes of social media use among our study participants did not provoke a physiological stress response but instead that sitting still and scrolling through content on smart phones likely depressed activity of both the sympathetic nervous system (as evidenced by a decreased heart rate) and the HPA axis (as evidenced by decreased cortisol)." We have added an additional sentence at the end of the conclusions to highlight needed future research: "More research is needed, to disentangle the physiological stress response from the psychological stress response, and emotional and behavioral responses in general, to more intensive periods of social media use." |
|  |  |
| Statistical Analysis The statistical analysis, including the use of a crossover design and measures of heart rate and cortisol, is robust. However, interpretation of the statistical results should be cautious, especially given the number of uncontrolled variables and possible variability in participants' individual experience with social networks. I suggest revisiting this point and drafting a paragraph that cautions these concepts. | In addition to the expanded text regarding exogenous stressors, we have added a very short paragraph about our limited subjects' representation of age and sex: "Lastly, because we used a convenience sample of participants, we may have had an inadequate number of teenagers and older adults participating, which may skew our results and conclusions. To adequately test the moderating effects of sex and age, a larger sample size across both variables may be required." We believe that these additions to the limitations, along with the unchanged text, speak to the full limitations and their consequences of the analysis. |
